# Supplementary material for: Setting the top 10 priorities for obesity and weight-related research (POWeR): a stakeholder priority setting process
Source: BMJ Open. 2022 Jul 20;12(7):e058177. doi: 10.1136/bmjopen-2021-058177 (PMC9305808; doi:10.1136/bmjopen-2021-058177)
Supplement: Supplementary data [file bmjopen-2021-058177supp001.pdf]

BMJ Publishing Group Limited (BMJ) disclaims all liability and responsibility arising from any reliance placed on this supplemental material which has been supplied by the author(s)

Supplementary Material

Supplementary Table 1: Organisations contacted to participate or distribute survey 1

Supplementary Table 2: Full list of 941 original questions submitted in survey 1

Supplementary Table 3: 149 Unanswered re-phrased questions in rank order from survey 2

Supplementary Table 4: Research questions for workshop

Supplementary Table 1: Organisations contacted to participate or distribute survey 1

Organisation contacted for distribution of survey 1	
DART	HomeStart
First Steps Nutrition	Oxford City Council
Food Foundation	National Obesity Forum
Good Food Oxford	Obesity Action Campaign
Guys and St Thomas'	HealthWatch Oxford
Local Government Association	Oxford Food Bank
NHS England	The All-Party Parliamentary Group on Obesity
Obesity Empowerment Network	Research For the Future
Public Health England	Activate Learning
SUSTAIN	Manor Surgery in Oxford
Obesity UK	Consultant in public health
Weight Watchers	Oxford Brookes
ASO	Kings College London, THIS institute Research fellow.
British Heart Foundation	Links with Obesity APPG
Centre for Food Policy	Department of Health & Social Care
Diabetes UK	Public Health Registrars
Eating Better Alliance	Commissioner of Healthcare Services
RSPH	Oxford BRC PPI
Weight concern	Newsletters
British Obesity Society	Department of PCHS
Obesity Policy Research Unit	Oxford Martin School
UK SBM	Blue Print
GP Nutrition Group	MedSci Division
BDA	Involvement Matters
LighterLife UK Ltd	Weight Management Panel
BritMums	
HOOP UK	

Supplementary Table 2: Full list of 941 original questions submitted in survey 1. 743 unanswered, 49 answered, 149 out of scope.

No. of questions	The 743 unanswered questions
1	What are the underlying genetic and physiological causes of obesity?
2	Further research on deepening understanding of factors which influence obesity on an individual basis (psychological, biological, genetic, etc.).
3	Connection between underlying health issues/genes/metabolic rates that may cause Obesity.
4	How much is obesity controlled by genetics, physiology etc. as opposed to a person's free will?
5	What are the underlying mechanisms for the journey to Obesity?
6	What factors are involved in weight related concerns - i.e. psychological/ psychosocial/ physical/ environmental? And which of these play a bigger part in obesity?
7	What are the social factors that contribute to obesity- and how can they be supported?
8	What are the social* and environmental determinants of obesity/body-weight? *this includes how social policies influence incidence
9	How do we tackle the upstream determinants of diet and physical activity related to obesity?
10	Fully understanding the reasons for obesity and factors with achieving weight loss.
11	What causes you to be obese or what do you think causes obesity?
12	What factors have influenced my weight gain?
13	Why do we gain weight?
14	What is the root cause of obesity and T2 diabetes?
15	What other than food affects your weight gain/loss?
16	What causes Obesity
17	How do genetics affect obesity?
18	My family has hereditary obesity issues. Will that affect me certainly?
19	Why do people become obese? Is it genetic? Is it just over eating?
20	What is the effect of genetics on body weight and on what BMI percentile somebody will fall in?
21	Research into is obesity passed from one from parents?
22	How can we combat the genetic component contributing to obesity- desire to eat/appetite, less ability to feel satisfied with food, preference for high calorie food?
23	Is obesity hereditary?
24	Is weight gain hereditary?
25	Is obesity hereditary?
26	I would like to know if genetics play a part.
27	Is obesity genetic? Can anything be done to prevent it?
28	How true is the public belief that they are predisposed to be overweight - the broad sweep statement that genetic factors are to blame for a huge proportion of the weight issues observed
29	Do genetics play a big part in obesity?
30	Are there inherited factors to obesity?
31	Is obesity genetic
32	How big a role does genetics play in familial obesity?
33	Why can some people eat what they want yet others put on weight when they do the same
34	What are the direct effects / mechanisms of poverty in relation to obesity
35	What are the impacts of poverty and socioeconomic status on health; on body weight; and on health related stigma? What policy decisions could effectively address and reduce the health disparity and life expectancy disparity between richer and poorer communities in Britain?
36	What are the key drivers in SES disparities regarding overweight/obesity?
37	What is the relationship between poverty and obesity?
38	Why are people from a lower socio-economic background more likely to have overweight or to be obese?
39	Is there a link with peoples income and them being overweight
40	Why is it that some people can eat what they like n not put on weight yet I only have to look at food and the scales go up.
28	How true is the public belief that they are predisposed to be overweight - the broad sweep statement that genetic factors are to blame for a huge proportion of the weight issues observed
29	Do genetics play a big part in obesity?
30	30. Are there inherited factors to obesity?
31	Is obesity genetic
32	How big a role does genetics play in familial obesity?
33	Why can some people eat what they want yet others put on weight when they do the same
34	What are the direct effects / mechanisms of poverty in relation to obesity
35	What are the impacts of poverty and socioeconomic status on health; on body weight; and on health related stigma? What policy decisions could effectively address and reduce the health disparity and life expectancy disparity between richer and poorer communities in Britain?

36	What are the key drivers in SES disparities regarding overweight/obesity?
37	What is the relationship between poverty and obesity?
38	Why are people from a lower socio-economic background more likely to have overweight or to be obese?
39	Is there a link with peoples income and them being overweight
40	Why is it that some people can eat what they like n not put on weight yet I only have to look at food and the scales go up.
41	Why we falter/fail when we know weight loss is beneficial
42	Why do some people gain weight when others do not when consuming similar calories?
43	Why do some people get fat and others don't
44	Why do some people gain weight and others don't on the same diet?
45	Causes of obesity
46	Why do some patients manage to keep significant weight loss off and yet others don't? Is it because of where their set point is? Is it genetic/epigenetic, or environmental or is it behavioural/psychological differences.
47	Who is most likely to recover from obesity, how and why?
48	Why some people don't put on weight even eating unhealthy and excessive food?
49	Why people in one family on much the same diet vary so much in weight?
50	Why can't some people lose weight?
51	are some people more prone to obesity, if so why
52	Which people with obesity will have a good response to behavioural weight management and which will need drugs or surgery to lose weight?
53	What subgroups of people with obesity respond differently to different behavioural interventions?
54	Why is weight loss maintenance so hard? [I am a serial offender. Have lost 3stones+ at least 3 times in my life (one of those was 5st) and 2stones+ at least twice.]
55	Why is it so hard to keep weight off after you lose it? [I've dieted many times and lost a lot of weight.]
56	Why do those empty fat cells shout 'feed me!' for at least 3 years after weight loss? Can anything be done to shut them up quicker??
57	Is the rise in obesity in the general public genetic or a learned pattern of behaviour
58	How much of an individual's weight is due to lifestyle factors, and how much is due to genetics?
59	How much of an effect is genetics and how much is environmental
60	How much of an impact does the environment have on an individual's weight (e.g. family circumstances, education, SES, availability and access to food), and is there anything that can be done to change this (government policies, retailer interventions, school food policies)
61	Why are certain ethnicities at a disproportionate risk?
62	Can we develop predictors of outcomes that can aid personalising treatment approaches?
63	What are predictors of weight loss success for a specific dietary or physical activity intervention?
64	What leads of healthy and unhealthy weight gain in children?
65	How can we make interventions close rather than widen gaps in deprived populations
66	How do I find information and solutions that apply specifically to me
67	Can we assess the cause of people's weight gain by simple questionnaire in order to target the most appropriate intervention? For example, those who are overweight would need different action, depending on whether the main contributor is a health condition (physical or mental), lack of exercise, financial constraints, making it difficult to buy healthy and filling food at low cost, busy lifestyle/lack of interest in cooking so that a lot of fast food is consumed, etc
68	How to study dietary preference in obesity management
69	What are the most effective ways to help people who are already obese manage their weight back to health, on an individual basis?
70	Why can't I get support for weight issues tailor made to my life style?
71	Can we subtype obesity by cause and use this information to offer more targeted interventions to prevent or reduce obesity?
72	With all the research into microbiome and the genetic responses to exercise is there a role for a more individualised approach to weight management?
73	For those who are found to have a genetic predisposition to obesity what can medicine do to prevent this undesirable outcome
74	Why do some gain weight in certain places on the body and not others?
75	Why does the fat go from where it goes from and which areas will lose first, etc?
76	What help would you like to see available, what help do you feel you need?
77	What are the benefits versus harms/risks of weight loss using different weight loss methods or specific types/groups of people? [If you are genetically pre-disposed to obesity, are you better to live at a higher weight, if trying to reduce your weight leads to repeated weight cycling] (Second part of question used in another section)
78	Are different diets right for different people? Or is simply that the best diet is the one you stick to? If some work better for certain types of person, how do you pick the one that would be best for you?
79	How can we personalise weight management?
80	How can we increase metabolism rate in old age, in order to decrease the chance of gaining weight
81	Does age have an effect on weight gain?
82	What is leading cause?
83	Are there times in people's lives that are associated with more weight gain and how can they prevent this?
84	As we age, do we need to consider consuming less food?
85	Why is it a struggle to lose the weight after middle age?
86	When did the individual become obese
87	How is your appetite
88	At what age does excess weight begin to adversely affect health?
89	Does the age at which people become overweight influence person's ability to lose weight later in life?
90	How long should an effective weight management intervention be to provide both weight loss and weight maintenance?
91	What is the lowest level of intervention needed for a response?
92	How can we better understand the effective components of behaviour change interventions

93	Is it not what you do but how many different things you do? I.e. the impact of whole systems…
94	What has worked for you in the past and why you could not maintain your progression?
95	What are your long term goals? Where would you like to be in 5 years' time?
96	Have you ever tried to lose weight before? How many times? And what was the outcome? - past experiences will help to find better solutions/option for a particular person
97	How can we maintain healthy behaviour patterns in relation to eating and exercising?
98	How can I maintain a healthy weight and lifestyle?
99	What characteristics of weight loss interventions are likely to lead to LONG-TERM MAINTENANCE of weight loss?
100	What are the evidence based interventions to maintain weight loss.
101	What's the best strategy to keep the weight off, once lost?
102	What is the most successful strategy to prevent weight regain?
103	Is there any method of weight loss that is reliable in the long term?
104	Once I lost some weight how avoid putting on again?
105	How to sustain weight loss or avoid increasing weight gain when obese if?
106	What are some of the most successful interventions in getting people to lose weight, and keep it off?
107	How to maintain the weight loss?
108	How can we ensure weight loss is sustained in the long term?
109	What is the best way to maintain weight loss
110	What is the best/most effective way of losing weight and keeping it off long-term?
111	How to achieve sustained weight loss
112	23. maintaining weight lost
113	What are the best regimes for long term weight management
114	How to prevent weight gain
115does	Having lost weight do you find it creeps back on and maybe even goes up?
116	Why do so many people who lose weight regain the weight they lost?
117	Research into keeping the weight off, once it has been lost as lots of people manage to lose weight and then put it on again.
118	Why do we slip back into destructive eating patterns so quickly?
119	What is the optimal dose of treatment contact required to prevent weight regain?
120	How can we maintain the modest weight loss achieved by lifestyle intervention or moderate weight loss achieved by VLED on the long run?
121	How can we apply a whole systems approach to weight loss maintenance?
122	Is there enough focus on transitioning from weight reduction to the maintenance of healthy weight?
123	Is weight maintenance feasible for any or many pts in weight loss programmes, and is this vital for health improvements
124	What is the best way to keep weight off in middle age?
125	What impact does it have on long term weight loss if the whole family is considered and not just the obese person in isolation
126	Identifying how much support is needed for weight maintenance
127	What creates success in weight loss? The dietary change/ choice or the long term support?
128	Research on services and messaging that focuses on behaviour change rather than weight loss
129	Which dietary strategies produce the most sustainable weight loss?
130	Why are weight loss programmes (weight watchers and slimming world) not effective in the long term?
131	Do we know which approaches to weight loss actually work?
132	Effective weight loss
133	What effective interventions could we deliver in primary care to help people with overweight and obesity
134	What Public Health programmes or interventions actually influence people to eat a healthy diet?
135	How do people lose weight easily?
136	How can I shift weight easily?
137	What works to reduce weight?
138	How to lose weight
139	Aside from restriction of calories and using more energy what else can I do to reduce my weight
140	For those who are already living with overweight/obesity, what are the most efficacious, cost-effective strategies and how can these be implemented?
141	How I can make changes in my diet so I can lose some weight?
142	Which weight loss methods are the most effective and long lasting? E.g. calorie counting, exercise, group support, portion control, food group's exclusion etc.
143	Has the person dieted, was this successful
144	Effective weight loss intervention development
145	How effective current treatments for obesity is m, are there any clinical treatments
146	How can people lose weight?
147	What is the most successful diet
148	What is an easy way to lose weight
149	How do I lose weight?
150	What is the best and correct advice to give to patients
151	Evidence based guidelines for weight loss
152	What actually is the best eating plan to follow it's a minefield out there so much advice on tv books etc. is there research that could prove the best eating plan to reduce weight then maintain it healthily
153	A holistic approach to weight loss
154	How can we help people change their lifestyles to maintain weight lost with very low energy diets?

155	How effective is schema-focused therapy in weight management?
156	How can we better engage people with weight loss services?
157	What could services do to encourage you to help yourself?
158	What can be done to help people stick to new healthy eating behaviours?
159	Why is weight loss maintenance so hard?
160	Why is it so hard to keep weight off after you lose it?
161	What are the best tools/technologies to help people adhere to long-term dietary and physical activity goals?
162	How do we get people to eat healthily and reduce obesity?
163	How to make lifestyle changes
164	What is a healthy lifestyle?
165	How to engage people (children and adults) who are overweight in weight management programmes
166	What are the barriers that prevent people from losing weight?
167	What stops you from losing weight?
168	What do people with obesity think is their biggest barrier to losing weight and keeping it off?
169	What are the real barriers in engaging the population in healthy eating for their children?
170	What are the cultural barriers to different BAME communities exercising in green spaces and what interventions can overcome these barriers.
171	How can we best help and support you?
172	What support do you need?
173	Repeated failure
174	Would you like to be a healthier weight?
175	What would you like to weigh/achieve
176	Do you want to lose weight?
177	'Willpower': Why can some people control what they eat whilst others cannot?
178	What is the motivation for your desire to lose weight?
179	Feel hopeless that can't get going
180	How to manage calories. Mind set to lose weight. Healthy eating. Why do we fail?
181	Why we falter/fail when we know weight loss is beneficial
182	What Weight management lifestyle Interventions can be effectively delivered in a primary care setting? Is there a place for group consultations in weight management?
183	Are slimming clubs effective
184	Would the client feel more supported if they had a weight loss buddy, who may also need to lose weight?
185	To make sure they have a social network so that they can mix with others doing arts and crafts etc.
186	Do you have good friends you can rely on, who would encourage you to lose weight, and motivate you? - It's good to have someone who supports you.
187	Is there a place for group consultations in weight management?
188	Is there a space for peer-support groups within weight management?
189	How effective are mobile applications in promoting weight loss among obese patients?
190	Can digital interventions improve both wt loss and weight management, and if so which ones
191	Can apps help you lose weight?
192	Do group activities help weight loss more?
193	How best to manage weight loss in housebound patients?
194	Does access to low-cost exercise equipment at home help with weight loss (i.e. mini pedals, which won't take up much room)
195	Exercise [how to exercise without having to go to a gym.]
196	What is the benefit of a weight-neutral approach to health
197	Consider shift towards behaviour rather than weight; review and include evidence that weight isn't directly related to poor outcomes - genetics & sociodemographic variables have a much stronger influence; effect of weight stigma on health; adopt health at every size approach.
198	How can weight-inclusive approaches to health be used to reduce stigma and better support improved overall health?
199	This could make the client feel more confident and it could prevent them from being lonely, which could affect their eating habits.
200	To constantly be alone at home is not healthy and can lead to bad eating habits and mental health issues.
201	Group consultations are becoming more popular and we would benefit from understanding where they have the most impact
202	Peer support has proven effective in other health conditions (e.g. stroke, diabetes, chronic pain etc.) in helping individual's access necessary support from those with similar lived experience. Ideas can be shared of how to cope with challenges that present with being overweight, and can reduce feelings of isolation (i.e. I'm not alone in struggling with my weight)
203	What role does social prescribing play in obesity?
204	Is there any evidence that social prescribing can benefit healthy eating?
205	Do BMI bands help to motivate the public to lose weight?
206	Who do people think should be responsible for their health? [Because it's important that health behaviour change messages come from credible/ acceptable sources]
207	Do you have to be thin (healthy weight) to discuss and treat excessive weight?
208	What can the food industry do to help the nation's palate develop healthier preferences?
209	Would subsidising fresh food lead to increased consumption and improved health? Would cost be offset by savings to NHS?
210	Research into Public health subsidised exercise and activity classes and their effects on rates of obesity in the respective communities?
211	If local authority swimming pools and gyms were subsidised or free would the cost be offset by improved health and lowered cost the NHS?
212	Which health policies that target the obesogenic environment (e.g. town planning, advertising restrictions, taxation) are most effective at reaching low socio-economic groups?

213	What can behavioural insights tell us about working to change the obesogenic environment? How can this complement the whole systems approach to obesity work?
214	How to change the environment so to make it less obesogenic e.g. fast food shops
215	How can we effectively change the obesogenic environment to promote adoption and maintenance of healthier diet and physical activity habits?
216	What specific strategies help to avoid temptation in an environment filled with food cues?
217	How does the obesogenic environment directly affect obesity rates?
218	Why, despite public awareness are there so many fast food outlets?
219	How can we change the behaviour of policy makers / town planners to restructure the obesogenic environment?
220	What is the impact of policies looking to change the built environment?
221	Implementation of healthy planning/ obesogenic environment
222	How can we change the environment to help prevent obesity? Should we implement more taxes, reduce advertising or do more?
223	How can we make environments less obesogenic?
224	Governance of food environment - thinking in systems
225	How can we adapt our environment to encourage less sedentary behaviour (through town planning/workplace policies/nudges etc.)?
226	Whether is it possible to navigate the obesogenic environment without gaining weight or whether the obesogenic environment is has to change?
227	What do we need to change in the environment to support people to have a health weight?
228	Which health policies that target the obesogenic environment (e.g. town planning, advertising restrictions, taxation) are most effective at reaching low socio-economic groups?
229	The obesogenic environment is a fairly new development, so why can't it be designed away again?
230	The impact on neighbourhoods built to promote active lifestyles on obesity and prevention of obesity.
231	How can better urban planning actually be implemented?
232	Modification of food environment
233	What makes people remain a healthy weight in an obesogenic environment?
234	How do we best prevent obesity in the first place; and how do we best lose weight over time if we have a few extra pounds?
235	How obesity can be *prevented*, rather than cured.
236	How to take sufficient,& sustainable preventative measures soon enough in a life to combat obesity
237	How to treat obesity and prevent it
238	What is best way to prevent, is it to never be obese or to fight it later?
239	Have any governments taken effective steps to tackle obesity, and could we learn from this?
240	What kind of public health promotion programmes and campaigns should governments be promoting/investing in?
241	More research into how other countries are managing to avoid having the same proportions of their population as overweight or obese.
242	What policy interventions can influence either obesity or other health outcomes related to obesity?
243	What kind of public policy changes are effective at helping people lose weight (e.g., traffic light labelling, calorie counts)?
244	What societal changes could be implemented easily / cheaply / at a local level - that would help reduce obesity
245	What community intervention strategies are successful for promoting weight loss in obese populations?
246	Do we have good evidence for community-based weight loss and management interventions?
247	What are the effects of population level or systems level interventions?
248	Is a wider public health rather than a targeted approach better of best used at the same time
249	How can we apply obesity prevention strategies at the population level?
250	What has research shown about if it is easier / cheaper to prevent obesity related diseases?
251	Early screening and intervention. Let's get creative, appropriate, and effective - what works?
252	Why doesn't medical intervention via paid subscription to a club or group start at 15% overweight, when it's still possible to see results and do exercise instead of waiting until 100%+ overweight, when exercise is impossible and loss so slow you ever see any end to dieting
253	What is the impact of the development and implementation of national food policies to support healthy weight in public settings such as schools and hospitals?
254	What influence does fast food advertising have on children obesity?
255	Should the government put heavy taxes on unhealthy processed food/drinks and heavily subsidise healthy organic whole food/drinks?
256	What is the impact of increasing sugar tax or other legislation to encourage healthier eating
257	How important are things like the sugar tax and food labelling in the prevention of obesity?
258	What is the widely held (including public) opinion on "fat taxing" or strict industry/commercial sanctions?
259	Should food and drinks manufacturers be given a "cap" for products (i.e. limited to so many calories, fat, sugar, etc?)
260	Any research connected to people living with obesity who are serving long term imprisonments?
261	How come side effects differ so much from person to person
262	We know that bariatric surgery is currently the most effective treatment for people with severe obesity (e.g. BMI > 40), yet most people with severe obesity don't choose this option even when it is available. Why? What options do people prefer?
263	What is the long term consequences of Bariatric Surgery exacerbate in those with an untreated Binge Eating Disorder?
264	How does Bariatric Surgery impact on intimate relationships with patients post-surgery? Exploring gender differences?
265	Do community health care practitioners know how to manage long-term post bariatric patients once discharged from acute care?
266	Are patients who have had bariatric surgery receiving the recommended follow up in primary care?
267	For individuals eligible for bariatric surgery, what aftercare support is provided and how long does this support last for?
268	Should dietetic and psychological resources be invested in preoperative setting or in post-operative settings?
269	Statistics to weight gain after so many years
270	What are the energy requirements with and without CVD and/or weight resistance exercise post bariatric surgery?
271	Although I don't eat much feel my metabolism lowered with band and gain weight very easy
272	What is the short and long term psychological impact of Bariatric Surgery?
273	How best to integrate exercise with commuting

274	Is active travel possible in both cities and rural areas?
275	What impact does active commute really have on weight loss?
276	How can we increase levels of physical activity for those working long hours where being sedentary is largely unavoidable?
277	Should employers provide benefits for staff e.g. gym membership discounts, walk/cycle to work schemes? How could this be funded?
278	What do people think about using standing desks at work? What would help motivate obese people to use a standing desk?
279	Do employers have an obligation to actively promote healthy lifestyles within the workplace to help employees with diet and obesity? Would this have an impact on absence levels and improve employee performance and engagement.
280	What more can employers do to help employees keep to a healthy weight?
281	How to fit exercise into your week
282	How can I add some exercise to my life?
283	What is the obstacles for people that stop them getting some physical activity
284	Why do obese people feel it is not possible to exercise? How can we make this very acceptable and applauded?
285	What could be done to encourage obese people to increase their physical activity?
286	Populations could include parents for school active travel, or adults travelling to work.
287	Whilst exercise causes us to produce endorphins our response to this varies from person to person, does having a lower response make us more susceptible to putting on weight and less likely to exercise?
288	How does people’s perceptions of air quality influence the likelihood of active travel?
289	Reduction in amount of activities
290	Do you get out of puff (short of breath) when you walk?
291	The importance of fitness/physical activity when overweight/obese
292	How can I better judge how many calories are burned by exercising?
293	How can we develop objective tracking of energy balance
294	Is there a phone app that can be used to photograph a person's plate / food that would provide information on the calorific and nutritional value of the food and inform the person when they had reached their daily recommended allowance. This allowance would be tailored to the individual in terms of age, sex, levels of activity and other health information.
295	Is there such a thing as an app which can accurately tell user what the calories are in a meal/drink, perhaps by using camera on phone and specific bowl/plate sizes (it can be very complicated if you have to input calories all the time)
296	Many interventions focus on changing people’s diets, but it is hard to measure what has changed without using long & detailed questionnaires or apps that often result in inaccurate results. Is there a way to measure diet quality that can easily be applied in clinical / research settings?
297	Do you lose more weight if you exercise or not?
298	Why do people think moderate amounts of exercise creates weight loss
299	Is there enough emphasis on combining healthy eating and exercise and a way of combating obesity rather than purely dieting?
300	How important is exercise in weight loss?
301	Is calorie controlling best way to control weight?
302	What is the best combination of dietary and physical activity strategies to maximize weight outcomes and appetite?
303	What is the best form of exercise to lose weight?
304	what is the most efficient exercise to lose weight
305	Most effective exercise
306	What types of sport contribute to a reduction in obesity for those that don’t regularly play or do sports.
307	What kind of exercise do I need to do and how often to lose weight?
308	Best forms of physical activity for weight loss/maintenance
309	What are the health risks of being overweight/obese but physically active? Does being physically active despite being overweight reduce disease risks of being overweight?
310	To what degree is higher weight due to more muscle OK vs higher weight due to more fat? Basically is it alright be to slightly overweight if it's 'all muscle'?
311	How can we widen access to effective weight loss support services for people affected by obesity?
312	Does regional variation in provision of obesity services have a positive or negative correlate with regional prevalence of - and trajectories in - obesity
313	How can obesity strategies be applied in practice?
314	What are the best early interventions that can be administered in primary care
315	Why can I get a gastric bypass on the NHS, but not Slimming World?
316	Do integrated lifestyle services offer equivalent outcomes and/or better value than individual programmes?
317	Should patients have direct access to Tier 3 services? Esp. those of high BMI
318	What is the effect of tier 3 weight management services on people with obesity?
319	How effective and cost-effective are level/tier 3 and 2 weight management services.
320	Are Tier 3 treatments better than Tier 2
321	Joined up services… we need research that creates a pathway from Tiers 1 - 4
322	Intervention Cost v Future Cost Saving for the NHS - To evaluate what financial (if any) benefits would be achieved for every £1 spent by the NHS on the interventions available to improve the health of people living with obesity.
323	Do economic evaluations based on modelling of cohorts over-estimate the benefits of weight loss? [Does weight loss return people to the same risk of those who never gained weight in the first place?] *Second part of question used in another section*
324	How can we develop better economic modelling for the impact of obesity and the benefits of its variety of treatments?
325	Does increase in public health funding reduce obesity
326	What is the effectiveness and cost-effectiveness in terms of preventing CV events (and diabetes and other consequences of obesity) for different weight loss / weight regain profiles?
327	We know that many people who lose weight will regain it. Are there long-term health benefits from short-term weight loss, and if so, is there a minimum amount of weight loss / duration that has clear benefits?

328	How to engage health service staff in recognising and supporting weight problems
329	What interventions are realistically delivered within the current NHS models of care e.g. nursing chronic disease reviews? How simple interventions or clinician training might impact. More of a focus on 1:1 interventions as well as Public health interventions.
330	What strategies can be used by GPs to promote physical activity in obese people?
331	Do doctors have the information they need to help all weight related issues?
332	What is the baseline knowledge regarding what a healthy diet consists of in the population? Then look at sub groups e.g. age groups, depressed, diabetes etc. Also research what doctors knowledge is of healthy eating AND how important it is to health, how much they feel confident to discuss this with patients or have time to.
333	How confident are health professionals when raising the issue of weight, and what is their current skill level to have these conversations
334	How can health professionals be more effectively utilised to support behaviour change conversations during routine interactions
335	Consultation skills: confidence and capabilities of health care professionals in raising the issue in any points of contacts
336	… Training and education requirements of different healthcare professionals to advice on nutrition on diet the role of pharmacists in weight and obesity management… *(Split - rest of question used in two other sections)*
337	How can we support implementation of obesity guidelines such that very person with obesity is offered significant support to reduce their weight with an acceptable chance of it working?
338	Is weight loss through diet or surgery of benefit for patients with type 1 diabetes?
339	Is weight loss surgery a good option for the severely obese, or would lifestyle changes/diet effect a similar long-term result?
340	Should the threshold for consideration bariatric surgery be lowered?
341	Is there any medication than can control appetite and suppress urges that is safe
342	Is there an effective satiety-inducing drug or other intervention?
343	About the design of drugs for obesity, should it focus on drugs for weight loss or should it focus on drugs not to recover lost weight?
344	Where do medications fit in the treatment pathway?
345	Design of a new obesity measure apart from BMI.
346	A new 'BMI' i.e. we know that BMI is often not relevant. Would be great to come up with a new 'scoring' system related to risk with weight
347	Why is BMI still used as an indication of healthy weight?
348	Is the BMI a good guide to healthy weight?
349	Why is BMI still used to define obesity?
350	With BMI being the crux of determining whether a person is overweight or not - is this notion now somewhat outdated?
351	Is BMI a reliable indicator of obesity, or not?
352	Why obesity seems somehow to be related to height but not frame size
353	To produce a better version of a weight measure than BMI
354	What better ways are there to assess weight other than BMI?
355	Accurate diagnostic criteria for obesity
356	Is our definition of obesity accurate
357	How accurate is a BMI as a guide to health if you are not an average body type?
358	What is the best way to estimate someone's healthy weight?
359	When will a more appropriate classification of obesity be made, focusing on the origin of the disease, which is necessary for a better treatment?
360	Is the BMI an appropriate way to decide if you're overweight for everybody, e.g., for BAME people?
361	How do I tell if I am a TOFI - Thin on Outside, Fat on Inside (i.e. around vital organs). I might be thin looking but have hidden fat issues.
362	The role of visceral fat [We are becoming increasingly aware that you can be 'skinny' but still unhealthy with regards to general health with high amounts of visceral fat.]
363	Clothing size could be used as a measure of weight.
364	What are the body composition changes post-bariatric surgery with and without weight resistance exercise?
365	What is the best, simple measure of adiposity in children?
366	Why is poor quality (highly processed) food so much cheaper than fresh/healthy food and how to get consumers to appreciate the health implications of each?
367	How can we make whole nutritious food more accessible and affordable than heavily processed foods?
368	What parts of the global food system are tending to increase the availability of unhealthy foods and what can be done to reverse these tendencies?
369	The effect on obesity if healthy food were cheaper and not healthy were very expensive
370	Decrease price of fresh food to enable people on low budget to stop eating processed foods.
371	Changing the environment in supermarkets and high streets - how can these organisations be motivated to improve quality of food choices
372	How can we make healthier food more accessible, attractive and convenient?
373	The effect of the availability of various foods on eating choices and how the current system encourages or discourages the production of "healthy foods"
374	How can we get nutritional advice/information to those most in need - e.g. low SES, obese
375	For people living with financial hardship, do they have access to information on how to eat more healthily for the same amount of money? This is often difficult to achieve. It would be interesting to see what difference education in this area made to outcomes for people and their families. Do people with unhealthy eating habits because of a lack of interest/time to cook, and a tendency to eat fast food, do better if they are educated in ways to make healthy changes that do not take much time and effort?
376	Health choices can sometimes be expensive can we do more to support cheaper locally sustainable options so that people can make healthy choices more partnerships with supermarket chains
377	Why is healthy food so expensive?
378	Pensioners and the widowed find it difficult to access cheap or food for one meals. How can this be addressed?
379	What can we do to make healthier food more affordable and appealing to young people?

380	The sale of baby foods high in sugars continues to be a factor in the obesity of children. How can producers be impacted to produce more healthy foods for children?
381	What research questions need to be addressed to support policy change which leads to stricter industry regulations on the size and composition of unhealthy foods available?
382	Research into whether government funded voluntary schemes for large retailers could help put better choices in front of consumers and change buying habits.
383	What impact does packaging have - for example the packaging of sugary yogurts vs plain yogurts in people's choices
384	Should products have a link to what exercise would be needed if you eat/drink a particular product (ie walking, running for 30 minutes)
385	What efforts are being made to identify sugar/corn syrup/harmful additives content in food? Why are harmful vegetable oils still being used, especially for take-outs? Can law be passed to embolden the content printing of harmful additives on containers? [How about advertisements/ doing further checks on school meals. Jamie Oliver did some great ground work. We need follow up.] *Second part of question used in another section.*
386	How is the degree of processing in the creation of pre-packaged foods best identified?
387	Should calories, fat and sugar in items be much more visible on labels and menus (traffic light)
388	How can we change social norms around the consumption of junk food / sugar?
389	The impact of reducing availability of energy dense foods on excess caloric intake
390	How can we encourage restaurants to serve smaller portions (or people to make smaller portions at home)?
391	How can restaurants help with portion control and calorie content of their meals / what impact does this have on obesity levels?
392	Have you heard of aspartame?
393	How does artificial sweetener affect weight gain or loss?
394	Do we understand the biology of how zero or low calorie sweeteners, particularly diet drinks, effect obesity
395	What role does artificial sweetener intake have on appetite regulation?
396	Do the huge amount of chemicals that are now used in food and drink production methods affect how the body's metabolism/hormones work, are these unpronounceable chemicals contributing to obesity?
397	Do you eat organic foods? [Do you know the benefits of organic & the harm of pesticides?]
398	Are the nutrient and energy levels compromised in GMO crops resulting in malnutrition?
399	Do GMO's contribute to obesity?
400	Do vitamin and mineral deficiencies cause or contribute to weight gain?
401	Natural ways to lose weight without dieting - how to lose the stubborn weight e.g. I only have 1 stone to lose not 10 but it's tough trying to lose it
402	Which is more important on the long run, calorie deficit or macro nutrients composition?
403	Do you see any link with diet and weight loss or weight gain
404	Does the keto diet cause greater weight loss than a non keto diet if the same amount of calories are consumed?
405	What is the right way to eat? High carbohydrate and low fat or High Fat and low carbohydrate? There is so much conflicting information. Why is the medical profession so conflicted?
406	What is more effective and healthy, low carbohydrate diets or low fat diets?
407	Does a low carb diet has long term side effects
408	Is a high fay, low carb diet a good way to stop putting weight on the stomach?
409	Is a well formulated LCHF diet healthy in the long term?
410	Is fat or sugar causing the obesity?
411	Is there a direct link between how much processed food is in a person diet to how many ailments may affect them later in life?
412	What is the impact on weight of eating processes foods? E.g. is there a correlation between eating processes food and increased calorie intake or weight gain.
413	Plant based eating for weight loss
414	Are plant based diets associated with obesity prevention, weight loss, and weight-loss management?
415	Is vegan a good way to reduce obesity?
416	How much meat should we eat?
417	Can being a vegetarian INCREASE weight?
418	Is it better to be vegetarian or eat meat fish etc.?
419	How to make all of us be more aware of the negative impact of high meat intake
420	Is protein from animal sources bad for our health
421	How much harm does a high protein diet do to your body?
422	Effectiveness of VLCD use in specific cohorts e.g. children/adolescents or pregnancy?
423	I've seen press on very low calorie diets. I'd like to know if they really work and, if they do, how to do them? Does it have to be under the supervision of a GP or can I simply buy meal replacement products at a pharmacy and get the same results?
424	How alcohol consumption influences weight gain.
425	Low calories versus low carbs?
426	Should we teach mindfulness and especially mindful eating to all? Could this make a difference to how we view food and eating?
427	We need more research into how meditation & self-care practices can reduce emotional (non) eating.
428	Can yoga help with weight loss?
429	Why is there no huge campaign about conscious eating? Slow eating? Learning to understand the feeling of hunger vs thirst
430	Do you often eat to the point of your stomach being uncomfortable?
431	Is there a way to turn on the "I'm full" switch earlier to avoid overeating
432	After my first diet I went on I got into a bad cycle of dieting or following a plan that doesn't work and end up binging or coming off the diet. I wasn't that big in the first place but family members were telling me I was Bonny!
433	What is the rate of eating disordered behaviour in people with obesity, particularly those who repeatedly try to lose weight
434	What percentage of people are overweight because of disordered eating (I.e. mental health)?
435	The calorific values of food. how can we accurately assess the amount we eat

436	Long term efficiency: Restrictive diets or permissive?
437	Is 'positive' messaging about dietary change (e.g. eat more fruit and veg, eat more fibre) more effective at maintaining a healthy weight than 'negative' messaging (e.g. eat less, fat, sugar, high calorie foods)?
438	How much weight and how fast is it lost using time-restricted eating?
439	Will intermittent fasting stop obesity
440	Fasting and if that helps lose weight and maintain it.
441	Does intermittent fasting work better than calorie restriction on its own?
442	Is intermittent fasting a good idea?
443	Is the intermittent fasting a truly healthy lifestyle to adopt?
444	Is intermittent dieting/fasting good for health beyond weight loss
445	Us fasting bad for your body? Either by restricting calories 2 days a week or eating in a 8 hour window
446	What thoughts on keto intermittent fasting
447	Do you know if you want to lose weight you should do intermittent fasting and you shouldn't eat carbohydrates?
448	How important is the timing of when you eat and what you eat?
449	The best hours (or routines) to eat.
450	How important is the practice of restricted time eating to weight control, metabolic health and well being
451	What is the impact of meal timing on weight, appetite and metabolic outcomes?
452	Late night eating (NE)
453	What foods should I avoid to help lose weight? [Should I be eating more protein after doing exercise and less carbs. it is quite confusing.]
454	Does the time between last meal at night and breakfast, the first meal in the morning have an effect on weight ?
455	Is okay to skip breakfast?
456	What impact does work hours (shifts / etc.) have on weight?
457	Which is better for weight loss, the traditional 3 meals a day or snacking throughout the day?
458	Is there really a difference between the different diets you read about in terms of diabetes remission? Can you get the same benefits with weight loss by any means?
See 336	Insulin resistance and its role in weight gain and obesity… *(rest of question used in two other sections)*
459	Are you aware of the role insulin plays in the storage of fat?
460	What other health conditions are directly impacted by obesity, as opposed to correlating with obesity?
461	Are fat cells created after puberty?
462	In the UK the shape of women's bodies has undergone a drastic change over the past couple of decades. What are the underlying causes, can we identify the people group, and how can we help reverse this?
463	Underlying issues when it comes to why people eat too much and or choose the wrong foods?
464	Look at why we eat from an environmental and behaviour perspective
465	Does my emotional state influences my overeating?
466	Are overeating behaviours linked to emotional states? Do we eat more when happy or sad?
467	How can I disassociate eating from how I am feeling?
468	Research into eating habits and mood swings, i.e. when do they binge. Trigger points.
469	Mental mind set how is this managed?
470	What is lacking (physically, emotionally or psychologically) when someone overeats and can we be taught to obtain this from another source?
471	Why are some people unable to not or stop eating foods they know are bad for their health?
472	Best methods to suppress appetites
473	How is the "I'm full now - you can stop eating" response triggered?
474	Should we start our weight management by sleeping more?
475	What happens in the brain during weight regain?
476	What parts of the brain can we turn off to control the addiction?
477	Is there an answer in neuroscience that actually physically disable the part of the brain responsible for the addiction? We do don't become addicted to alcohol / drugs etc. so young because we are not exposed to them, but we are exposed to food, and the pattern is set.
478	Impact of Autism on eating patterns
479	Does stomach muscles have a direct link to satiety? If you have loose stomach muscles due to pregnancy , will you not feel full how much ever you eat
480	Energy Requirements for pre and post bariatric patients at various stages after surgery (e.g. 6mo, 12 mo, 18mo, 24mo)?
481	How do changes in body weight and composition influence the energy balance behaviours that may undermine weight management interventions
482	Diagnostic algorithm for cardiovascular risk stratification in patients with obesity
483	How much does the microbiome affect absorption and which foods (if any) can keep it at its optimal level
484	The mechanism behind the effect of the gut microbiome in obesity development.
485	To look at the microbiome of level 3 weight management services and see if they carry particular obesity genes and to see what the diversity of their gut microbes look like.
486	How does the microbiome influence body habitus - using metagenomics could contribute to this question
487	Can faecal transplant impact weight
488	Does healthy bacteria in the intestines affect /reduce weight gain?
489	Can improving gut bacterial alongside dietary/ exercise interventions help increase weight loss and maintain weight loss?
490	How can we change our gut microbiome to help maintain a healthy weight
491	The best forms of pre- and pro-biotics to improve health and facilitate weight loss.

492	What effect does GOS supplementation happen on the adverse outcomes of obesity
493	Can Obesity cause or contribute to Cancer and Dementia?
494	To explore the relationship between the over-consumption of antibiotics and the increase in obesity.
495	Can some antidepressant meds cause weight gain;
496	Which, and by how much, do prescribed medications adversely affect the patients weight?
497	Impact of medications on obesity
498	What is the best way to don't put weight while taking sertraline?
499	The effect of obesity on other health issues including dementia, arthritis etc.
500	Does losing weight reduce the risk of cancer and how much weight do you need to lose
501	Does weight loss reduce risk of heart failure in people with known heart failure?
502	Do patients with mild/moderate heart failure benefit from weight loss interventions?
503	Do obese people have more digestive problems than normal weight people?
504	Weight loss plan for those with IBS and Diverticulitis and gluten intolerance
505	Does one specific type of diet work for a single disease (e.g. a vegan diet for cvd)?
506	Relationship between obesity and osteoarthritis (if any)
507	How obesity affects bone health
508	Impact on joints
509	What impact does maintaining a healthy weight have on mobility (knee joints etc)?
510	Knee wear
511	Hip wear
512	In older adults exercise can be difficult due to rheumatic issues. How should diet accommodate this
513	Is it better to aim to be slightly underweight to reduce stress on the joints in old age?
514	The relation between long term illness and weight gain.
515	Pain and Obesity, eating as an avoidance strategy for pain?
516	Why do women with polycystic ovaries often put on a lot of weight
517	What effects do hormonal changes have on weight and how can one differentiate between gain due to hormones vs diet.
518	What level of BMI reduction is required to significantly increase the chance of ovulation for obese women with amenorrhoea as part of PCOS?
519	How effective is weight loss as a treatment for PCOS?
520	What is the effect on weight gain of the removal of half of a thyroid gland and 16 months later a hysterectomy with the removal of ovaries?
521	Under treatment of underactive thyroid: is there a need to review current guidelines and treat 'subclinical' hypothyroidism?
522	Do you have any medical condition, e.g. thyroid problems, which might cause obesity?
523	Do you feel you have become obese as a result of medical / physical condition or some other factor
524	Why have I had a life long struggle with my weight, despite not over eating [I have only recently found out I have lipoedema. If I had known this earlier, things might have been different.]
525	What motivates and facilitates people with chronic illnesses who are overweight or obese to lose weight? Which biomedical and socioeconomic barriers do people face, that prevent them from being able to lose weight and what can be put in place to overcome these obstacles?
526	What effects does it have on the heart?
527	If my type 2 diabetes is in remission (blood-sugar levels 5.4 or lower), am I still diabetic?
528	Does the amount of sugar eaten worsen liver disease
529	Is red meat bad for liver disease
530	What is the impact of diagnosing non-alcohol related fatty liver disease as a motivator for people to lose weight
531	What evidence is there that children and teenagers post liver transplant will find it challenging to maintain a healthy weight?
532	Are obese people more likely to get colds
533	Why do some men have man breasts [People suffer mental health issues due to this]
534	How much does obesity limit and impact our life
535	What impacts does obesity have on health
536	The role in obesity in relation to prevention of ill health? [Because prevention at an early stage will help improve the quality of life for a vast number of people]
537	By how much does being obese affect your predicted life span
538	What is the life expectancy of obese people as compared to similar individuals who are not overweight?
539	What is the impact of obesity on peoples' lives including their life opportunities?
See 77	What are the benefits versus harms/risks of weight loss using different weight loss methods or specific types/groups of people? [If you are genetically pre-disposed to obesity, are you better to live at a higher weight, if trying to reduce your weight leads to repeated weight cycling] (*First part of question used in another section)*
See 323	Do economic evaluations based on modelling of cohorts over-estimate the benefits of weight loss? [Does weight loss return people to the same risk of those who never gained weight in the first place?] (*First part of question used in another section)*
540	The physiological impact of early menopause and weight gain.
541	Understanding of why women put on weight before menopause when never previously struggled with weight
542	What is the effect of hormones during and after menopause on weight gain
543	Why is weight loss so hard post menopause?
544	I've now come through the menopause and it is even more difficult to maintain a steady weight.
545	Heavy/abnormal periods and whether they are weight related or not
546	Hormones/hormonal process. How they affect? Are these changes real? How could we counteract effects? Hormone injection for weight loss?

547	The relationship between testosterone levels and visceral fat?
548	We know there is a relationship between high levels of visceral fat and low testosterone levels, and this is also associated with higher CVD risk. Is the converse true? Are higher levels of testosterone associated with lower levels of visceral fat and/or overall CVD risk
549	What impact do male/female hormones have on appetite/weight/metabolism?
550	Does a baby in utero have a higher risk of becoming obese if Mum is obese during pregnancy?
551	How targeting prenatal development can reduce obesity risk
552	How can we prevent young women gaining excessive weight, including during and between pregnancies.
553	What is the effectiveness of weight management programs in the postnatal period and does this lead to a reduced BMI in subsequent pregnancies?
554	Weight increase in pregnancy
555	What is the most effective method to reduce gestational weight gain in obese women?
556	We're you breastfed as a baby? [As a mother who has struggled with breastfeeding but has persevered due to the many health benefits, I would like to know more and to have better research on the impact of breastfeeding and weaning on obesity later in life.]
557	Does educating school children about how to make healthy eating choices, prepare nutritious food quickly, and shop for healthy food at a low cost make a difference to their overall health and weight in the long term?
558	Does educating children within schools on healthy eating help prevent obesity?
559	Do you think if prevention was started in primary school your outcomes would have been improved?
560	How to prevent obesity in young people and inform at schools?
561	What more can be done at a primary school level to tackle obesity?
562	Are children educated about this topic
563	How to avoid childhood obesity and help child stay a healthy weight
564	What are the most effective health promotion interventions to halt weight increases in children between the ages of 5 and 11?
565	Which specific factors within health promotion interventions to promote healthy weight on school aged children are effective?
566	Why/how/what has had an impact on the areas where we are seeing a reduction in childhood obesity?
567	How can we reduce childhood obesity and make sure children grow up with healthy diets?
568	What are the most effective interventions for the prevention of childhood obesity globally and are they implementable in the UK?
569	Weight in children
570	Policy to promote healthy lifestyle and physical exercise from early age
571	What age does healthy eating need to be established in order to prevent adult obesity?
572	What works in preventing childhood obesity? What works to help people maintain a healthy weight? What works long term to help people lose weight? The influence of mind over body in obesity?
573	Childhood obesity prevention
574	How can childhood obesity be prevented?
575	How to prevent obesity in young people and inform at schools?
576	Should counselling be provided to school children who are overweight?
577	Maintenance of a healthy diet, with group counselling all the way through school.
578	Should schools place a higher priority on nutrition education and cooking skills?
579	Would increasing the provision of home economics e.g. cooking skills, budgeting skills, reduce the incidence of adult/childhood obesity
580	Would better nutrition/cookery courses throughout a child's WHOLE school life make a difference to their understanding of healthy eating?
581	What are the essential elements of school food education programs that help children learn positive food behaviours?
582	How can we educate/promote sensible choices from childhood when sugar is everywhere? Would this make more sensible adults?
583	How can effective school food education programs be leveraged to influence the home environment?
584	How do we encourage parents to establish healthy eating habits in their kids?
585	Are parents educated to the consequences of bad eating habits in their children?
586	There is a need for greater funding for personnel and locations for education of parents regarding healthy nutrition. How can this situation be reversed and made more of a priority?
587	How to help families with children under the age of 11 avoid their children putting on too much weight
588	How can we best support children and families to change long term behaviour for weight loss
589	How do we engage parents in meaningful conversations to raise the topic of overweight/healthy growth in an effective manner?
590	What is the best way to encourage children to eat healthily?
591	How can we most effectively engage with an adolescent population regarding diet & exercise?
592	Diet and weight in adolescents.
593	How best to engage teenagers in exercise programmes?
594	What can we do to keep children active into adolescence and adulthood?
595	Would 1 hour a day of physical activity / sport per day in state schools lead to improved health and obesity levels in children and adolescents and improved habits and health in to adulthood? (97% of school population in the state sector. Independent schools already spend more time doing sport than state schools.)
596	Strategies to prevent weight gain young adult
597	What support is best for obese teenagers?
598	How to prevent childhood obesity - particularly in UK ethnic minorities and deprived populations.
599	How do we iron out the social gradient in obesity prevalence, particularly in children?
600	Children between 1 year and 4 years are found to be overweight by health visitors. They do not qualify for a healthy weight and exercise programme until they are 4. What are the best ways of managing these children?
601	Practical education and will help confront the problem and bring resolution. Group therapy is effective amongst a group of people who experience great social difficulty.
602	How to encourage Healthy meals uptake in school settings
603	Is adult obesity related to parental attitudes to food and eating?

604	Can we improve coded weight/height/BMI data for children in the GP record? I have an interest in Childhood obesity and would be interested in considering process that would help to code weight related data in the primary care record. There are currently over 27,000 children aged 0-16yrs with registered GP practices in Oxford alone, of these only 19 children had recorded BMI >25, yet we know through NCMP data the prevalence of higher weight status far exceeds this, plus we know very few contacts with families through current programmes for tackling this (e.g. 2019 NCMP only generated 4 referrals in Oxfordshire)
605	What are the current effects of the National Childhood Measurement Programme in terms of weight trajectories, health care usage, and mental health?
606	Is NCMP fit for purpose if, as in many places, it is to become a screening programme/pathway into child weight management?
607	What are the repercussions of obesity in childhood, in later life?
608	Does obesity impact on children's long term educational outcomes?
609	Does obesity impact on children's long term life chances?
610	How does obesity impact on children's happiness, confidence and mental health?
611	To explore the relationship between adverse childhood events (ACES) and obesity at a population level.
612	The relationship between ACE and obesity (either childhood development or adult).
613	Did you have an easy/stress free childhood?
614	Do childhood or adolescent negative events have an impact on unhealthy eating?
615	Is there a connection with mental trauma in childhood to obesity?
616	We need more research into the role of childhood trauma in causing obesity and / or weight related issues in adulthood.
617	Childhood trauma and being morbidly obese
618	What treatments for obesity should be available to children/adolescents?
619	What effective management options are there for paediatric obesity - who should people be referred to/supported by? And how widely available/accessible are these currently?
see 748	… Is there any improvement in the figures for schools which provide lunches/boarding schools? (*First part of question answered see 748)*
See 385	[What efforts are being made to identify sugar/corn syrup/harmful additives content in food? Why are harmful vegetable oils still being used, especially for take-outs? Can law be passed to embolden the content printing of harmful additives on containers?] How about advertisements/ doing further checks on school meals. Jamie Oliver did some great ground work. We need follow up. (*First part of question used in another section)*
620	How to get the rest of your family on board with supporting in your efforts to transition to a more healthy routine
621	How do participatory approaches with families within the community setting influence implementation and influence on healthy weight behaviours?
622	Parents often do not engage with healthy weight programmes because they deny their children are overweight. How can parents be more active in recognition of overweight children?
623	How much is related to socially and family dysfunctional lives
624	How do we assess and monitor diet quality in infants, older children and adolescents in a valid, robust, consistent and pragmatic way?
625	How can I make quick and easy meals with minimal effort so that I lose weight?
626	Can you cook
627	Benefits of local communities teaching basic cookery skills
628	Encouraging people to grow healthy foods
629	Has the plethora of cookery related programmes on TV had any impact on the nations diet or are they pure entertainment, unrelatedto what/how people eat
630	Are images of obese people in dieting information sheets motivating or demotivating.
631	Do food choices based on environmental and sustainability concerns influence weight status in young people?
632	If we change the words and conversations we have around weight and bodies and food, could this be the biggest difference we n eed in battling the emotional, mental and physical damage done to us by over or under eating, and society's obsession with the body perfect?
633	What are the consequences of weight stigma on physical health
634	How can we combat obesity stigmatisation and discrimination?
635	How can we reduce weight stigma and discrimination in the society?
636	What can be done to change society's attitude towards obese people?
637	What terms could be used instead of obesity to describe the policy area?
638	What are you doing to prevent people being/feeling shamed because of their weight?
639	How can we reduce obesity stigma?
640	How can we reduce the stigma of weight regain?
641	10. What communication is needed to change public perception of Obesity?
642	What are the effects of weight stigma?
643	How should stigma around obesity be dealt with?
644	How can we combat weight stigma?
645	Help to address the stigma around weight and weight loss
646	How can public health messaging about overweight/obesity be delivered to maximise effectiveness and minimise stigma?
647	Do GPs assume that obesity is the fault of the patient rather than something to do with food marketing and/or healthier food availability for poorer people?
648	What can we do to shift an entrenched view within our society (including among some healthcare professionals) that those living with overweight/obesity are entirely responsible for their condition?
649	Is there an institutional bias against people with obesity in NHS England, trusts, CCGs and the country in general?
650	Can we reduce weight bias in health professionals (GPs, nurses, dietitians)?
651	Do health staff treat obese people differently, and less favourably, than those of recommended weight?
652	How can we reduce health inequalities for obese people, wrt prejudice they may face from medics, whether conscious or unconscious?
653	Do GPs assume those with lower BMIs (20-25) are somehow healthier (or more likely to be healthy) than those with higher BMIs?

654	Why aren't people living with obesity given the same consideration as others with chronic diseases?
655	Are overweight people less likely to receive appropriate health investigation as obesity is seen as causative first, rather than following the same diagnostic route that you would immediately follow with a person whose weight is considered normal/acceptable?
656	Why are people who are obese not given the same access to healthcare? People who are obese are blocked from receiving NHS surgery by virtue of their weigh. Why is the NHS discriminatory against obese people in the supply of surgical procedures?
657	Does obesity stigma influence healthcare professional referral practices
658	Is access to primary care compromised for people classified as overweight or obese because GPs tend to focus on that rather than the concern/symptom the patient actually presented with?
659	Explore whether how all health care professionals are trained around obesity perpetuates weight stigma attitudes which are then reflected in their delivery of care
660	What words used in a GP consultation influence a person's desire to change their eating habits? (Evidence based)
661	How can GPs tackle consultations regarding weight loss in the most sensitive manner? Should the emphasis be on diet as opposed to weight at all?
662	Are you happy with your weight?
663	I, as an individual, have been received as better thin than fat.
664	Do current BMI bands and definitions make sense to the public?
665	What forms our image of a healthy body and how can this be used to promote a healthy weight and how society be educated in what is a healthy body weight?
666	Why is obesity accepted as a norm and not treated as a serious problem?
667	Is society's acceptance of obesity creating a feeling in the obese that it is okay to neglect yourself in the way?
668	Is the body positivity movement ok? Is it ok to celebrate morbidly obese body shapes?
669	What role does constant media talk of the obesity crisis or 'drain on NHS have on weight bias and perpetuating obesity?
670	How does the internet, and specifically social media, affect the drive to eat?
671	Does social media influence eating behaviours?
672	What role has the media played in causing obese people to have low self-esteem?
673	How much is obesity related to education and personal responsibility
674	Why don't people who are obese make an effort to lose weight? Is it a lack of dietary understanding; poor motivation: or acceptance of their body image?
675	Why do people continue to be obese, when we have plenty of evidence to tell people how not to be?
676	Is it a mind-set problem that causes people to be obese and not deal with it?
677	What role should personal responsibility play in obesity discussions?
678	Why is there not more emphasis on personal responsibility when it comes to weight control?
679	Why don't people take ownership of their own health practices
680	What is the link between depression and being overweight
681	How does being overweight make you feel?
682	Mental health and obesity
683	The psychological reasons that trigger obesity
684	What is the psychology behind obesity?
685	How would you relate your weight to you mental health?
686	Mental Health issues, probably also relates to question above
687	Correlation of mental health issues with obesity.
688	Totally life changing after being depressed about weight gain
689	How does obesity affect mental health?
690	What causal mechanisms link the social experience of obesity to poor mental health outcomes?
691	Have you considered obesity as a mental health issue, as well as a physical one?
692	Is there a non-physiological trigger for obesity (such as a psychological trigger like bullying which lead to comfort eating)?
693	Is there any correlation between mental health illness and obesity?
694	The physiological impact of stress and weight gain.
695	What are the effects of diet culture and a focus on weight loss on individuals' mental health?
696	What are the physical and mental health effects of repeated dieting
697	How have past diets impacted on my health?
698	What is the impact of repeated dieting on health?
699	Understanding the impact of repeated attempts to change lifestyle habits e.g. is there a parallel with quit attempts
700	How does others perspectives on a person's weight impacted their mental health?
701	Why are there so many physical, mental and emotional implications from being overweight?
702	Why is obesity detrimental for people?
703	What's the effect of obesity on self confidence
704	Over-giving to others as an defence against low self-esteem and driver of obesity
705	How do you feel as a fat person How do people view you as a fat person Do fat people smell Are thin people better than fat people
706	How does obesity affect social behaviour and self-esteem?
707	How does being obese impact social interactions, and how obese people feel they are perceived?
708	We need more research into how, if we help individuals identify and understand how they are feeling, that aids a reduction in emotional (non) eating.
709	Can interventions that address emotional or social overeating be effective?
710	Does therapy help to eliminate emotional eating l
711	How to break the habit of using food for comfort.
712	What mental health/emotional support best helps a person living with obesity?
713	Overcoming psychological barriers to weight management - can this lead to weight loss when the focus is not on weight?

714	How can the psychological component of obesity be addressed?
715	Are self-image, self-care and self-confidence sufficiently taken into account in current weight reduction and healthy weight programmes?
716	Mental health…why is this so important when losing weight? Would a diet based in mental support only be as effective?
717	To look at the impact of trauma focused therapy upon weight loss
718	Research further: Eating is used as a psychological avoidance strategy to avoid contact with past trauma which causes beliefs /feelings of "I'm not ok or I'm empty" and as a way of avoiding experiencing present feelings and negative thoughts?
719	How to maintain good mental health when struggling with your physical health - in this case, obesity.
720	To ask when the client first started eating more food.
721	How do I get surgery on NHS my dr says Im depressed and my health suffering
722	How should weight management services and pathways be optimised for populations with serious mental illness?
723	The link between obesity and depression is well known. Yet why is it, in the absence of thorough investigatory medical care, antidepressants are prescribed to obese people without explanation that these drugs will contribute to weight gain?
Part of q 336	…Weight gain and obesity in severe mental illness - how do we manage this? *(Question used in two other sections)*
724	Do you eat when stressed?
725	How does stress relate to weight gain
726	Are you stressed? Do you think you are on top of things in your life? What is work like?
727	Tackling stress to affect obesity
728	How can we reduce or prevent the impulse to eat in people with obesity?
729	Is sugar addictive?
730	Helping obese people psychologically to overcome food addiction.
731	How the addiction to food becomes an issue so young .
732	How do you stop food addiction
733	How can you turn off the sugar cravings?
734	Treatments to modulate hedonistic behaviours - salt intake, confectionery intake, etc
735	What does sugar do to your body and why do I crave it?
736	Does sugar make you fat?
737	Understanding of body treating all carbs as sugar. Seems hard to believe that bread and a chocolate bar can both be treated by the body as sugar
738	How to minimise late night comfort eating
739	Why do some people crave carbohydrates and do some people react differently to certain carbohydrates.
740	Do you eat when you aren't hungry?
741	Why do people eat when they're not hungry? What is the link between emotion and consumption? Is there a biological driver?
742	How unhealthy eating, mental health and alcohol misuse interplay. What is the most effective way in to tackling this?
743	Can overeating be seen as a type of self-harm?
	**49 Answered original questions (5.2% of all submitted questions)**
744	Obesity vs waist circumference and waist to hip ration for a marker of CVD in adolescent men
745	Has the decline in teaching Food and Nutrition in senior schools had an impact on the diet of society?
746	Why aren't we teaching children about proper nutrition in school? Nutrition that is based on the latest research, not 30-40 year old findings.
747	Is there a link between social deprivation and childhood obesity?
748	Which groups of kids are most likely to be obese in school (socio economic background, ethnicity, gender, etc.), how does this correlate with their mental health and performance…*(second part of this question (not written here) is unanswered and has been taken forward)*
749	Do weight loss initiatives and diets actually work? What are the associated risks?
750	Carbs, calories, low fat, high starch, syns, points, there must be one method that actually works, without vast expense, thinking of how many have to rely on cheap food, and leaves you not hungry all the time.
751	Will eating more fruit and veg make the energy last longer
752	Does drinking water help you lose weight?
753	What is being done to work with industry to reformulate recipes to be less calorific?
754	How obesity affects fertility
755	impact on fertility?
756	Why do men lose weight much quicker than females? Is this something that research may discover something that could help females?
757	What are the best (short - medium) outcomes to show the effectiveness of weight management in pregnant women?
758	How has pregnancy and have a child affected your weight?
759	The effect of rapid weight loss on your health
760	Is being underweight better than being overweight?
761	The rise in bowel cancer and obesity- is there a causal effect or just a correlation?
762	Is being over weight linked to cancers - which ones?
763	Need to emphasis the importance of rapid weight loss in ending diabetes.
764	Relationship between obesity and diabetes?
765	Diabetes
766	Diabetes and nutrition, and how to prevent or reverse type 2
767	How much weight loss is needed to prevent those at high risk developing T2DM?
768	Type 2 diabetes now gone
769	Does obesity cause, rather than is it associated with, adverse outcomes?
770	How does obesity affect your health?

771	What evidence is there for the ways in which weight affect various disease processes?
772	What is the best why to avoid obesity related disease?
773	How much weight gain is permissible before I am at a risk of diseases?
774	What are the five most common health complications that arise from obesity.
775	How does weight impact on chronic illness and function?
776	What is the link between obesity and illness/disability?
777	Related medical condition
778	relationship between obesity and longevity - and is there a genetic tendency to obesity
779	Genetically are we becoming inbred?
780	what are risks relating to gastric band
781	What single change would make the biggest difference to weight loss
782	What is the best second line treatment for late gastric dumping post surgery?
783	Failure of procedure
784	Complications of procedure
785	Are psychological interventions being offered routinely within weight management services in the UK?
786	How does stigma around weight and obesity contribute to difficulties initiating weight loss?
787	What impact does weight stigma have on those trying to lose weight?
788	How mental attitudes impact on people's ability to lose weight
789	Weight stigma and the experience of the overweight/obese
790	Weight-based stigma in healthcare professionals: how this impacts people's ability to lose weight
791	What is the correlation of certain amount of physical activity (run, walk, etc.) with the calories we burn?
792	Would psychological counselling regarding eating behaviours make people more likely to loose weight and maintain it?
	**149 out of scope submissions (15.8% of all submitted questions): out of scope / not a research question / too broad / not obesity related / opinion based / unclear/ unclear but similar question in included research question above.**
793	What is your correct weight bearing in mind BMI
794	How do I find out what is normal and healthy for my age and gender?
795	How is obesity measured?
796	Why do you think you are overweight?
797	Should obesity be considered a disease?
798	Staging of obesity
799	What is Obesity? Specific definition and subtypes.
800	How to encourage Healthy meals uptake in school settings.
801	How much damage to our bodies and how they work do fad diets actually achieve? Are we not just chasing an expectation that doesn't exist?
802	How to navigate and choose the best diet type?
803	Do you know the three macronutrients?
804	How can I get past health information on food products to understand what is bad in them, not just what is good?
805	healthy diet promotion and education
806	Why is sugar so freely available?
807	Why, as consumers, are we sold unhealthy food to eat? If trans-fatty acids are as bad for our health as is now being said why is the medical profession, the government and the authorities not informing people? Why are they not preventing the sale and banning the production of processed foods containing theses fats?
808	What are the corporate determinants of obesity/body-weight?
809	How do we get government to implement more effective policies regarding selling and producing unhealthy food etc.
810	How does obesity policy fit into wider public health policy?
811	When was the latest guidance on nutrition in obesity published and who recommended/reviewed it.
812	What is the current and best guidelines to use
813	How much natural, non-processed food do you eat?
814	The only downfall is the excess saggy skin Is it possible to be 'overweight' but still in good health?
815	Does a 'healthy' BMI always reflect healthy physiology? Can you be overweight and healthy?
816	Can one be healthy at any weight?
817	My parents are both type 2 diabetic, lifestyle-induced. Does this mean I am certain to be as well?
818	Do you have any medical / physical conditions
819	Should obesity be recognised as a disease?
820	Is it calories in and then exercise means calories out ?
821	How can we bring more 'eating behaviours' interventions into standard practice?
822	Do we need to focus on body weight or risk factors/health?
823	Lap band had 12 years ago worked but broke last feb had removed had bypass in june in latvia at much expense dudnt work
824	will there be a miracle cure in the future?
825	Is there a "cure" for obesity?
826	What are the psychological contradictions for Bariatric Surgery?

827	Should there be more psychological assessment available for those with obesity other than those with diagnosed eaing disorders?
828	Post operative psychological advice is lacking. This is only open to specific individuals? Other than funding, why has this not be identified as an integral part of the bariatric process
829	Would psychological counselling regarding eating behaviours make people more likely to loose weight and maintain it? I am about to start a program of counselling as a pre requisite for bariatric surgery. I have also had experience with Overeaters Anonymous. I feel that half the battle when it comes to obesity is mental & emotional.
830	Can obesity now be labelled as an eating disorder as it is without a doubt disordered eating and results from many if the same complex psychological, emotional and behavioural issues as anorexia, bulimia etc and can have equally devastating results mentally and physically.
831	Psychologically informed services, training etc
832	Do fat people need to feel bad about themselves?
833	Do you enjoy food
834	To ask the client how they think they would feel if they were able to lose weight and what they miss doing the most.
835	Do you require and emotional or psychological help or guidance?
836	Do you think you would be happier if you were not obese?
837	Are you happy with your size
838	Can increased metabolism rate means you can eat more food
839	How do you speed up your metabolism?
840	On a scale of 1 to 10 (with 10 being the happiest), how happy are you with your weight?
841	What would make it a 10?
842	What could we do to help you make it a 10?
843	What could you do to help you make it a 10?
844	What do you already understand about obesity and weight management?
845	to change diets and attitudes to food
846	Does shaming obese people really work
847	Why are the aids via drugs which are available in the USA ignored in the uk
848	Are there safe drugs available to treat the condition?
849	Is there a magic pill that you can take to lose weight?
850	What level / amount of physical activity / exercise do you undertake in an average week
851	What is you weekly routine in terms of "movement": how often do you exercise, do you walk to work - if yes how far, do you walk up the stairs or use the escalator and so on, if you have a dog, do you take it for a walk and how long are those, how busy is you after work life: do you have to manage the kids or do you come home and sit in front of the TV.
852	Does the person do regular exercise to manage weight
853	Do you do any exercise?
854	What can we learn from the epidemiology of obesity to help in terms of prevention?
855	Do you think it's possible to lose weight and would you like help to lose your excess weight.
856	Why if you ever manage to lose weight, having wasted your life in fat, does the nhs refuse to assist with skin removal, thereby giving you a body to be proud of instead of a grotesque thing to hide, which will inevitably be refilled
857	Do you know what services are available to support weight loss?
858	The type weight-loss programme that is suitable dependent upon size, age and physical health presentation. To reduce potential risk of injury, stroke or heart attack
859	Are researches into obesity funded by government?
860	Who should help patients lose weight? What is the evidence that things such as MECC actually works? Or should we leave it to professionals with obesity treatment experience?
861	The cost to the NHS for adapting specialist equipment
862	What kind of help do you feel that the NHS could provide?
863	How do you feel about your weight?
864	Does malnutrition (diet of poor nutritional value) increase the risk of obesity
865	What food combinations best help you to lose weight?
866	Crisps or not
867	Have you drunk any fruit juices?
868	What is the truth about milk?
869	Butter and full fat milk ?
870	Successful interventions for disorders which lead to weight loss
871	Xxxxxxx
872	Is there as much time/money spent on obesity-related diseases as those related to being severely underweight?
873	Re-formulate how weight with other factors is 'healthy' and 'unhealthy'
874	Can we identify at an individual level what rate of weight loss is optimal to avoid harmful complications such as excess skin, gallstones etc
875	How and best to support our most difficult patients
876	Success rate for each procedure
877	healthy lifestyle initiatives at Primary Care level
878	How strong/reproducible is that evidence?
879	How can guidelines best communicate uncertainty and variability to the public?
880	What is the impact on the offspring of mum having hyperglycaemia in pregnancy?

881	Is the DASH diet effective in reducing the risk of preeclampsia in those with gestational hypertension?
882	Impact of changes in ingredients and preparation methods on the nutritional quality of food consumed.
883	Is our food poisonous to us?
884	We need more research into how counselling can help identify new strategies to reduce emotional (non) eating.
885	Eating Disorders eg anorexia can be devastasting for those affected, and those who surround them - more research to alleviate needed
886	Is it more likely to suffer from an eating disorder if you've had a life saving procedure?
887	Is veganism a form of eating disorder?
888	Understanding the underlying psychological and physiological mechanisms of eating disorders
889	How do you feel about your body?
890	How can eating disorders affect the absorption of anti-rejection medication?
891	What foods should you regularly eat?
892	What is the healthiest diet
893	no 5 How can we ensure more people (incl. children) consume a high-quality diet (vegetables, fruits, beans, etc)? why? National and international survey data suggest that people have suboptimal diets.
894	Diet
895	Any new guidance and tips
896	What are the most common misconceptions about dieting and weight-loss?
897	What can / is being done to tackle the sheer amount of fake news, fads and terrible advice when it comes to nutrition and diets.
898	How can we educate people and get support from the food industry to get more info about the right food.
899	How do dietary patterns and food choices based on environmental and sustainability concerns influence weight status in young people?
900	How to prevent the escalation in childhood obesity in low and middle income countries
901	What lessons that we have learnt in the UK can be communicated and employed in "developing" countries where the next epidemics of obesity are happening?
902	What can we learn from the obesity problem in developed countries that can help prevent the same problems in the developing world?
903	What foods actually contribute to increased weight and obesity ?
904	What foods do you blame for your obesity?
905	What food can help break down fat?
906	Health
907	understanding the obesity paradox in health of certain patient groups and older patients . Is intentional weight loss always beneficial for health
908	Type2 Diabetes is common in older adults. How can this be avoided?
909	what food to eat
910	Using systems dynamics modelling techniques, what are the optimal intervention points on the Foresight Obesity Map?
911	What research is there into the link between post transplantation patients and anorexia?
912	How to eat sustainably?
913	Exercise
914	More active and healthy
915	Health
916	Increasing Age and weight [impact on NHS]
917	Is weight the best proxy for the health impact of weight management programmes?
918	What is your average daily/weekly calorie intake (intake recorded)
919	Why calory restriction diets do not work?
920	What do you eat?
921	What do you normally eat and drink on a daily basis?
922	Am I on a healthy diet?
923	Do you know what balance diet is and do you think you have one?
924	What has/has not worked for you previously?
925	How much research is being done on the subject of nutrition?
926	Why isn’t there a fast food chain that just do healthy food?
927	Is governmen nutritional advice wrong
928	How sure can I be that things like 'five a day' and alcohol units are based on solid findings and not just arbitrary figures in the general direction of better health.
929	Why are the most effective treatment for obesity (bariatric surgery) is underutilised in the UK?
930	When will a true multidisciplinary treatment be available, especially with psychological / psychiatric treatment, for patients with obesity?
931	Adjusting mental attitude needed to achieve weight loss.
932	is there a way we can be tested to find out what foods are best suited to our metabolism
933	Do you personally think obesity is a health issue?
934	Am I taking enough exercise
935	I have a sweet tooth and throughout the day, I usually consume three to four chunks of sweetmeat or chocolates. Will that increase my chances of obesity definitely or just a probability?
936	How can natural experiments be best used to evaluate the potential of obesity interventions? (methodological)
937	I imagine that other studies have been conducted on this topic. Will data from these studies be taken into consideration, along with your own (comment)

938	How can governments be influenced to adopt preventive health measures around obesity rather than focus on treatment? (policy/ implementation)
939	Obesity prevention at the population level: where does political appetite align with evidence? (policy/ implementation)
940	How can we speed up translation of promising findings from early trials on how to prevent or treat obesity to the real world? What sources of funding or NHS 'pipelines' would help? Could money from the sugar tax or similar be used for this? (about dissemination)
941	What factors/ characteristics are shared by people who love being active and how does these characters differ from those who hate activity?

Supplementary Table 3: 149 Unanswered re-phrased questions in rank order from survey 2

Rank	Re-phrased questions	Mean score (SD)
1	What are the most effective methods for weight maintenance following weight loss? What are the effective components of treatments/ programmes incorporating a behavioural element? How many and in what combination are most effective? What is the optimal duration of these programmes?	8.36 (1.87)
2	What is the cost and affordability of a healthy balanced diet? What policy measures could make healthier foods more affordable? What policy measures could improve access to healthy diets for different social and cultural groups, such as people in poverty, people in inner cities, or young and older people?	7.85 (2.06)
3	Does an intervention that focuses on improving overall health rather than a specific focus on weight loss improve health and wellbeing?	7.81 (2.25)
4	Does having a psychological disorder such as depression and anxiety affect weight, and, if so, what is the mechanism? How do external perceptions of weight affect mental health? What is the effect of weight loss methods and repeated dieting on mental health outcomes?	7.75 (2.03)
4	What are the most effective methods for weight loss? What are the effective components of treatments/ programmes incorporating a behavioural element? How many and in what combination are most effective? What is the optimal duration of these programmes?	7.75 (2.36)
6	What is the most effective and cost effective mix of population/ public health and individual interventions to tackle obesity?	7.72 (2.26)
7	Do interventions (e.g. nutrition education and physical activity) in pre-school, primary school and secondary school reduce children's risk of unhealthy weight gain and, if so, how do they act? Does the effect of such interventions differ by social and cultural groups?	7.67 (2.22)
7	What are the drivers of food choice, appetite, and intake and do variations in these drivers explain who develops obesity and who does not?	7.67 (2.24)
9	What are the most effective ways to prompt people to make a weight loss attempt or engage with a weight loss support programme? What are the most effective methods to increase uptake and adherence to programmes?	7.65 (2.30)
10	How do demographic, social and cultural factors, e.g. age, socioeconomic status (SES), lifestyle, environment, psychosocial functioning affect weight status, weight gain and regional fat distribution? What are the mechanisms involved? Does the effectiveness of weight loss methods depend on social and cultural background and, if so, can the effects be made more equitable? Are weight loss methods tailored to people's background more effective for weight loss and weight maintenance than general methods?	7.59 (2.41)
11	Are people living with obesity at higher risk of infectious diseases? Are they at higher risk of severe consequences from these diseases? What are the proposed mechanisms?	7.54 (2.31)
=12	Is weight loss an effective treatment for certain medical conditions and chronic illnesses, e.g. polycystic ovary syndrome (PCOS), hypothyroidism, lipaedema, gastrointestinal conditions, heart failure, osteoarthritis, chronic pain etc? Should specific weight loss methods be recommended for people with overweight and obesity with certain medical conditions and chronic illnesses?	7.53 (2.40)
=12	Do parents' attitudes to food influence their children's food intake and risk of obesity?	7.53 (2.35)
=12	Does the quality or amount of sleep influence bodyweight? Do interventions that increase/ decrease sleep affect weight loss and weight maintenance?	7.50 (2.15)
=12	In people living with overweight and type 2 diabetes, does losing weight lead to diabetes remission and how long does remission last? What health checks are needed for people who have achieved diabetes remission?	7.47 (2.41)
=16	Does losing weight and regaining it repeatedly adversely affect long-term health or psychological wellbeing compared with staying at a higher weight the whole time?	7.45 (2.60)

=16	How do genetic factors, physiological and metabolic processes affect weight status, weight gain and regional fat distribution? What are the mechanisms involved? Do these factors affect the ability to lose and maintain body weight? Do weight loss programmes tailored to a person's genetic makeup or physiology produce greater weight loss than untailored programmes?	7.45 (2.20)
18	Do interventions that target the 'obesogenic environment', such as community interventions, urban planning, placement of fast food outlets or workplace policies, affect population mean weight and do these effects differ by baseline weight status (underweight, healthy weight, overweight, obesity)? Which of this type of interventions are most effective at reaching low socio-economic groups?	7.44 (2.40)
19	Why do some people struggle to maintain motivation during a weight loss attempt?	7.41 (2.54)
20	What are the effects of obesity in childhood on outcomes other than physical health e.g. psychological wellbeing, educational achievement, employment, relationship status etc?	7.39 (2.37)
21	Does incorporating physical activity into weight loss interventions enhance weight loss? Does this depend upon the frequency and type of physical activity (e.g. high vs low intensity, resistance vs cardiorespiratory activity)?	7.36 (2.37)
22	What effect do working hours (e.g. shift work) and/or a late eating pattern have on body weight and adiposity?	7.35 (2.70)
23	Does being physically active mitigate the health consequences of overweight/obesity?	7.31 (2.12)
23	Does prolonged psychological stress affect bodyweight? Do interventions that aim to reduce stress increase weight loss in people living with overweight and obesity compared with interventions not addressing stress?	7.31 (2.20)
25	Do interventions that address emotional or social overeating lead to weight loss or weight loss maintenance?	7.29 (2.43)
26	What is the relationship between emotions and appetite or energy intake?	7.19 (2.29)
27	What changes in supermarkets or the wider food industry are effective in promoting healthier diets? Does changing labelling and/or packaging on foods affect purchasing, consumption and body weight?	7.18 (2.36)
28	Do interventions in primary care prevent obesity? What are the effects of offering weight management referrals on the prevalence of obesity?	7.17 (2.30)
28	How accurate are existing models of the health consequences of excess weight and the impact of weight loss? Which assumptions are critical in determining the long-term effectiveness and cost-effectiveness of weight loss interventions? What is the impact of weight regain on the incidence of disease and cost-effectiveness of weight loss interventions in these models?	7.17 (2.21)
28	How does the brain control food intake and can we use knowledge of these mechanisms to aid weight loss? What are the brain responses to food during weight loss and following weight regain?	7.17 (2.51)
28	Does social influence affect the amount of energy dense-nutrient poor foods or consumption of healthier foods, and can these norms be changed?	7.17 (2.24)
32	What is the effect of parental support for their children's weight management on weight in a child living with obesity? Are family based interventions aiming at childhood weight control effective?	7.16 (2.34)
33	What are the effects of interventions to influence health professionals to support patients with overweight and obesity?	7.15 (2.32)
34	What is the effect of adult obesity on social behaviour, self-confidence, self-esteem and self-worth?	7.14 (2.38)
35	Does the gut microbiome have an effect on weight gain or the risk of obesity? Does the gut microbiome influence body composition or fat distribution? Do human genetics influence the gut microbiome? Can a change in gut microbiome aid weight loss and by what mechanisms? Which strains of bacteria are associated with greatest weight loss during a weight loss attempt? What is the effect of prebiotics or probiotics on weight loss/weight maintenance?	7.12 (2.53)
36	What impact does obesity have on life opportunities? E.g. employment, marital status, happiness, education.	7.11 (2.65)
=37	How effective and cost effective are subsidies for healthy food or access to leisure facilities to prevent or treat obesity?	7.10 (2.45)
=37	Does obesity cause dementia?	7.10 (2.34)

=37	Is there a difference in the way that health professionals respond to patients living with obesity compared with people of a healthy weight in terms of investigation, diagnosis and follow up?	7.10 (2.49)
40	Are interventions to treat children or adolescents living with severe obesity effective?	7.08 (2.54)
41	Is it better to advise incorporating physical activity into daily life or taking formal exercise to increase overall levels of physical activity in the population?	7.07 (2.41)
42	Are there benefits of some types of diets on diabetes that are independent of the amount of weight lost?	7.06 (2.27)
43	What is the impact of reducing the availability of energy dense-nutrient poor foods on overall energy intake?	7.05 (2.77)
=44	What are the mechanisms that lead to satiation during meals?	7.04 (2.28)
=44	Does having a choice of how to lose weight improve the success of the weight loss attempt?	7.04 (2.48)
=44	In people living with obesity, does losing weight affect how the heart works?	7.00 (2.32)
=44	How do we assess cardiovascular risk in people living with overweight and obesity? Should we stratify interventions for weight loss based on cardiovascular risk in those living with overweight and obesity?	7.00 (2.24)
48	Can the provision of healthy school food reduce obesity in children?	6.99 (2.73)
=49	What are the most effective treatments for obesity in people with severe mental illness? Is integrating weight management into mental healthcare effective in supporting weight loss or weight loss maintenance?	6.98 (2.36)
=49	Does living with obesity affect quality of life? Does losing weight or gaining weight affect quality of life in people living with overweight and obesity?	6.98 (2.61)
=51	Do family dynamics cause unhealthy weight gain?	6.97 (2.59)
=51	Why do some people living with overweight and obesity do insufficient physical activity and what advice or interventions might increase this?	6.97 (2.52)
=53	Do interventions to encourage healthier food purchasing affect population mean weight or the prevalence of overweight and obesity?	6.96 (2.46)
=53	Does eating a diet high in processed foods make losing weight, maintaining lost weight, or gaining weight more likely? Do diets high in processed foods lead to a higher incidence of weight-related illness, such as diabetes or heart disease, independent of body weight status?	6.96 (2.69)
=53	Does eating a diet high in processed foods make losing weight, maintaining lost weight, or gaining weight more likely? Do diets high in processed foods lead to a higher incidence of weight-related illness, such as diabetes or heart disease, independent of body weight status?	6.96 2.69
55	What are the health risks for individuals who have previously been overweight but have now lost weight compared with those who have never been overweight?	6.93 (2.43)
56	How do energy requirements change following weight loss and what are the mechanisms involved?	6.92 (2.36)
57	Can interventions modify individuals' food preferences and does this affect weight outcomes?	6.91 (2.29)
58	Does the imagery used in weight loss interventions and public health messaging affect motivation for behaviour change?	6.88 (2.48)
59	Does the amount of sugar in baby food affect children's weight gain?	6.83 (2.62)
60	Is overeating a form of self-harm for some people living with obesity?	6.80 (2.86)
=61	Does the mode of delivery e.g. in-person face-to-face, group, digital, telephone influence the weight loss achieved by a weight loss programme?	6.80 (2.45)
=61	Does age of onset of obesity or years lived with obesity influence the effectiveness of specific weight loss methods?	6.80 2.27

=63	Does childhood trauma or mental health disorder in childhood cause obesity?	6.79<br>2.63
=63	Is attempting to avoid snacking more or less effective than 'little and often' for weight loss and weight maintenance?	6.79<br>2.56
65	Does fast food advertising affect the prevalence of overweight and obesity in childhood?	6.78<br>2.80
66	Do markers of blood glucose control and cardiovascular disease (e.g. insulin, glucagon, HbA1c, blood pressure, cholesterol etc) predict weight gain and obesity? What effect do these markers have on weight loss and weight maintenance?	6.77<br>2.39
67	What are the most accurate and practical ways to measure dietary intake?	6.76<br>2.67
68	Do weight loss interventions improve blood glucose control or influence cardiovascular risk in people living with type 1 diabetes and obesity and what are the adverse effects?	6.75<br>2.51
69	Is there institutional bias towards people living with overweight and obesity in different employment sectors and can this be changed?	6.73<br>2.72
70	Do diets high in fat or high in sugar increase the likelihood of gaining weight?	6.71<br>2.80
=71	What are the effects of tier 3 services on weight loss and are these services cost-effective?	6.68<br>2.20
=71	Do restrictive or permissive diets lead to greater weight loss or weight loss maintenance? Is permissive messaging about dietary change more effective at maintaining a healthy weight than restrictive messaging?	6.68<br>2.26
73	How do the words used to define and describe overweight and obesity affect perceived discrimination as well as emotional and psychological wellbeing in people living with overweight and obesity? Would changing the language reduce the blame culture associated with obesity? What language used in public health messaging about overweight/obesity encourages weight control without increasing stigma towards people living with obesity?	6.67<br>2.75
=74	What is the role of the adipocyte life cycle in body weight and obesity?	6.66<br>2.36
=74	Do food additives and non-nutritive sweeteners affect appetite regulation, post-prandial metabolic and endocrine responses and energy intake? Is replacing sugar with non-nutritive sweeteners an effective method of weight loss and weight maintenance?	6.66<br>2.70
76	What medical conditions can lead to weight gain and obesity?	6.65<br>2.55
77	Do social prescribing schemes such as community gardening, cookery classes, choir, lunch club, or book clubs lead to greater weight loss when trying to lose weight than attempting weight loss without such prescription?	6.64<br>2.47
78	What effect does social media have on eating behaviours and weight control?	6.63<br>2.61
79	Are there methods to combine pharmacological and behavioural weight loss treatments that mean the combination is greater than the sum of the parts?	6.62<br>2.69
=80	What is the prevalence of disordered eating in people living with obesity? Do weight loss interventions increase the risk of disordered eating?	6.61<br>2.22
=80	Is it possible to suppress appetite and so reduce energy intake? Is this an effective way to lose weight?	6.61<br>2.68
82	Can screening programmes identify those at risk of developing obesity? Does identifying people at risk and implementing early interventions prevent weight gain?	6.59<br>2.67

83	What is the role of social networks in weight control? Does having a weight loss buddy lead to better outcomes?	6.58<br>2.41
=84	What is the effect of behavioural, dietary, pharmacological, and surgical interventions on body composition and fat distribution? Can exercise attenuate loss of lean tissue associated with weight loss?	6.57<br>2.31
=84	What interventions can help parents to identify if their child is overweight and does this increase their engagement in weight management services?	6.57<br>2.67
86	What are the advantages and disadvantages of the various methods of quantifying body fat in routine healthcare? What is the association between these measures and risk of obesity-related health outcomes? What are the most appropriate classifications for overweight and obesity in different demographic groups, e.g. ethnicity, age, gender? Can we develop an easy measurement for 'Thin on Outside, Fat on Inside' for use in routine healthcare? What is the association between being 'Thin on Outside, Fat on Inside' and other obesity related outcomes?	6.55<br>2.34
=87	Do interventions in the general population to improve cookery skills improve weight control?	6.53<br>2.53
=87	Is limiting daily energy intake a more successful method than limiting daily carbohydrate intake for weight loss and weight maintenance?	6.53<br>2.52
=89	Is attempting to follow intermittent fasting (including 5:2, every other day diet, etc.) or time restricted eating (e.g. 16:8 or eating between specific hours of the day) effective for weight loss and weight maintenance? Is intermittent fasting or time restricted eating more effective than calorie restriction alone? Are the benefits from intermittent fasting or time restricted eating (e.g. metabolic health, general wellbeing) independent from weight loss? Are there any side effects or health risks from intermittent fasting or time restricted eating?	6.52<br>2.60
=89	Do people crave or become addicted to certain foods and, if so does this cause obesity? Can treatments reduce pica, food cravings or addiction?	6.52<br>2.53
=89	Is taxing unhealthy foods and drink effective in reducing the prevalence of overweight and obesity?	6.52<br>2.81
92	Is obesity a cause or a consequence of reduced physical activity?	6.51<br>2.54
93	Would rules that limit the energy or macronutrient content of certain categories of food products affect the prevalence of overweight and obesity?	6.49<br>2.54
=94	Does the menopause cause weight gain/loss? Does the age at menopause affect weight gain and weight loss?	6.46<br>2.46
=94	What effect does social media have on self-confidence, self-esteem and self-worth of people living with overweight and obesity?	6.46<br>2.78
=96	What are the most accurate and practical ways to measure total energy expenditure and physical activity?	6.44<br>2.66
=96	In women entering pregnancy with overweight or obesity, do intentional efforts to reduce the amount of weight gained reduce the risk of obesity in the offspring?	6.44<br>2.59
98	Does informing a person living with overweight or obesity that she or he has non-alcoholic fatty liver disease (NAFLD) motivate weight loss?	6.40<br>2.60
99	Are there long-term health benefits from short-term weight loss? If so, is there a minimum amount of weight loss/ duration that has clear benefits?	6.39<br>2.39

=100	Are interventions aimed at weight control effective in helping women entering pregnancy with overweight or obesity limit weight gain during pregnancy or lose weight after birth?	6.38<br>2.47
=100	Are total diet replacement programmes with behavioural support as effective as using diet replacement products without behavioural support?	6.38<br>2.66
102	What are the effects of the body positivity movement on body weight?	6.36<br>2.70
103	Does breastfeeding increase post-partum weight loss or reduce risk of later obesity for the mother?	6.34<br>2.70
104	What are the effects on health of having a body mass index (BMI) within the overweight/obese range yet with high proportion of lean tissue/high muscle mass?	6.33<br>2.62
=105	Has the National Childhood Measurement Programme had an effect upon weight trajectories, health care usage, and mental health of children or their parents?	6.32<br>2.67
=105	Does attempting to limit alcohol intake reduce the risk of weight gain or increase weight loss?	6.32<br>2.75
107	Does the low carbohydrate ketogenic diet cause greater weight loss than a reduced carbohydrate non-ketogenic diet? Is attempting to follow a ketogenic or reduced carbohydrate diet safe and effective for weight loss and weight loss maintenance? What are the effects on fat distribution/body composition of these diets? Are there any adverse effects or long-term health risks?	6.31<br>2.88
108	Does the fact that most people are overweight mean that people are less concerned about their own excess weight?	6.28<br>2.76
109	Are workplace interventions to support active commuting, or schemes to increase physical activity provided through workplaces feasible, acceptable, and do they affect weight?	6.26<br>2.23
110	Is training people to eat less through techniques such as mindfulness or slow eating, or responding to internal hunger and thirst cues effective in helping people lose weight?	6.25<br>2.76
=111	Do some people living with overweight and obesity not recognise that they are overweight and what effect would interventions to improve recognition have?	6.19<br>2.65
=111	Do interventions, including clinical follow-up, after bariatric surgery prevent weight regain?	6.19<br>2.56
=111	Does the perception that the causes of obesity relate to matters of personal responsibility motivate people to control their own weight?	6.19<br>2.49
=111	What are the adverse consequences of bariatric surgery and can these be predicted?	6.19<br>2.51
115	Do marketing campaigns or policy changes affect population mean weight and do these effects differ by baseline weight status (underweight, healthy weight, overweight, obesity)? Do marketing campaigns, policy changes and community interventions prevent weight gain, prompt attempts to lose weight or enhance weight loss in those attempts?	6.18<br>2.52
116	How effective are interventions to reduce portions in restaurants, café/ food outlets on energy intake and the prevalence of overweight and obesity?	6.16<br>2.77
117	Do the prevailing messages about the costs of obesity on healthcare affect prevalence of obesity or bias against people living with overweight?	6.15<br>2.66
118	Does the level of naturally occurring sex hormones (testosterone, oestrogen, progesterone) influence bodyweight? Does administering sex hormones lead to weight loss or weight loss maintenance, e.g. Hormone replacement therapy (HRT) , testosterone etc?	6.14<br>2.43

119	Are high protein diets safe and effective for weight loss and weight loss maintenance?	6.10<br>2.65
120	Does the macronutrient composition of the diet influence the progression of non-alcoholic fatty liver disease (NAFLD) in people living with obesity?	6.06<br>2.53
=121	What methods are available to measure diet in infants, children, and adolescents and how do they compare in reliability and practicality?	6.02<br>2.68
=121	Is the use of commonly prescribed medications (e.g. antibiotics and pain medication) related to weight gain and obesity? What are the effective methods to prevent weight gain and achieve weight loss in people taking medications known to cause weight gain?	6.02<br>2.43
123	How effective are emerging pharmacological treatments for weight loss and how can we develop new drug treatments to help people lose weight (e.g. appetite suppressants, nutrient absorption etc)?	5.99<br>2.71
124	Why do some people with overweight try to lose weight and some people with overweight do not?	5.98<br>2.64
125	Do interventions in the general population to grow food improve weight control?	5.91<br>2.64
126	Does having heavy or irregular periods affect bodyweight?	5.89<br>2.56
=127	Are total diet replacement programmes (based on energy restricted formula food products) effective, safe, and tolerable for weight management in special populations e.g. attenuation of weight gain in pregnant women or for other groups where rapid weight loss may change outcomes, such as prior to a planned surgery?	5.86<br>2.55
=127	Why do people living with severe obesity eligible for surgery decide not to proceed with the surgery and do they take up other interventions?	5.86<br>2.55
=127	Does endorsement or delivery of interventions by different sources enhance the effectiveness of weight loss interventions? E.g. GP referral, celebrity endorsements, social media, family and friends. Does measurement and acknowledgement of body mass index (BMI) by a credible source influence motivation to attempt weight loss or adherence to weight loss programmes?	5.86<br>2.51
130	Is a vegan, vegetarian diet, pescatarian diet or a diet inclusive of meat better for weight loss, weight maintenance and metabolic health?	5.83<br>2.67
=131	Has the fat distribution in females changed over time?	5.82<br>2.80
=131	Does effectiveness of weight loss interventions vary depending on weight status of the individual delivering the intervention?	5.82<br>2.66
133	How do diets with varying macronutrient composition affect adherence to energy restriction?	5.80<br>2.72
134	Do vitamin and mineral deficiencies cause or contribute to weight gain? Do vitamins, minerals and other dietary supplements aid weight loss?	5.70<br>2.52
135	Does variation in gastric capacity explain who does and does not develop obesity?	5.63<br>2.46

136	Does advice to omit breakfast lead to greater weight loss and better weight maintenance than dietary advice that includes breakfast?	5.60<br>2.75
137	What is the effectiveness and cost-effectiveness of bariatric surgery for people with type 1 and type 2 diabetes with any degree of overweight but with a body mass index (BMI) < 35?	5.57<br>2.54
138	Do concerns about the environment and sustainability affect weight?	5.55<br>2.78
139	What is the impact of cookery related television programmes on diet and weight?	5.48<br>2.84
140	Are people with addictions to drugs and alcohol at greater risk of unhealthy weight gain?	5.39<br>2.73
141	What are the psychosocial consequences of bariatric surgery for example, on intimate relationships and eating disorders, and do these vary by social and cultural group?	5.32<br>2.45
142	What are the methods available to measure adiposity in children? How valid and reliable are these methods?	5.31<br>2.48
143	Does pseudogynaecomastia in men living with obesity influence psychological wellbeing?	5.23<br>2.52
144	Does autism increase the risk of obesity?	5.16<br>2.55
145	How does people's perception of air quality affect their engagement in outdoor physical activity and does this influence their weight?	4.80<br>2.74
146	Does having a liver transplant in childhood or adolescence lead to unhealthy weight gain?	4.67<br>2.42
147	What is the association between clothing size and body mass index (BMI)?	4.66<br>2.92
148	Do 'speciality foods' including genetically modified and organic foods influence total energy intake and hence bodyweight?	4.56<br>2.76
149	How does imprisonment effect body weight? What factors influence weight change and obesity during imprisonment?	4.46<br>2.49

Mean number of people who ranked each question (SD): 115 (9.7) Mean priority score (SD): 6.61 (2.5)

Supplementary Table 4: Research questions for workshop

Survey 2 top 30 debated at the workshop

1. What are the most effective methods for weight maintenance following weight loss? What are the effective components of treatments/ programmes incorporating a behavioural element? How many and in what combination are most effective? What is the optimal duration of these programmes?
2. What is the cost and affordability of a healthy balanced diet? What policy measures could make healthier foods more affordable? What policy measures could improve access to healthy diets for different social and cultural groups, such as people in poverty, people in inner cities, or young and older people?
3. Does an intervention that focuses on improving overall health rather than a specific focus on weight loss improve health and wellbeing?
4. Does having a psychological disorder such as depression and anxiety affect weight, and, if so, what is the mechanism? How do external perceptions of weight affect mental health? What is the effect of weight loss methods and repeated dieting on mental health outcomes?
5. What are the most effective methods for weight loss? What are the effective components of treatments/ programmes incorporating a behavioural element? How many and in what combination are most effective? What is the optimal duration of these programmes?
6. What is the most effective and cost effective mix of population/ public health and individual interventions to tackle obesity?

7. Do interventions (e.g. nutrition education and physical activity) in pre-school, primary school and secondary school reduce children's risk of unhealthy weight gain and, if so, how do they act? Does the effect of such interventions differ by social and cultural groups?
8. What are the drivers of food choice, appetite, and intake and do variations in these drivers explain who develops obesity and who does not?
9. What are the most effective ways to prompt people to make a weight loss attempt or engage with a weight loss support programme? What are the most effective methods to increase uptake and adherence to programmes?
10. How do demographic, social and cultural factors, e.g. age, socioeconomic status (SES), lifestyle, environment, psychosocial functioning affect weight status, weight gain and regional fat distribution? What are the mechanisms involved? Does the effectiveness of weight loss methods depend on social and cultural background and, if so, can the effects be made more equitable? Are weight loss methods tailored to people's background more effective for weight loss and weight maintenance than general methods?
11. Are people living with obesity at higher risk of infectious diseases? Are they at higher risk of severe consequences from these diseases? What are the proposed mechanisms?
12. Is weight loss an effective treatment for certain medical conditions and chronic illnesses, e.g. polycystic ovary syndrome (PCOS), hypothyroidism, lipaedema, gastrointestinal conditions, heart failure, osteoarthritis, chronic pain etc? Should specific weight loss methods be recommended for people with overweight and obesity with certain medical conditions and chronic illnesses?
13. Do parents' attitudes to food influence their children's food intake and risk of obesity?
14. Does the quality or amount of sleep influence bodyweight? Do interventions that increase/ decrease sleep affect weight loss and weight maintenance?
15. In people living with overweight and type 2 diabetes, does losing weight lead to diabetes remission and how long does remission last? What health checks are needed for people who have achieved diabetes remission?
16. Does losing weight and regaining it repeatedly adversely affect long-term health or psychological wellbeing compared with staying at a higher weight the whole time?
17. How do genetic factors, physiological and metabolic processes affect weight status, weight gain and regional fat distribution? What are the mechanisms involved? Do these factors affect the ability to lose and maintain body weight? Do weight loss programmes tailored to a person's genetic makeup or physiology produce greater weight loss than untailored programmes?
18. Do interventions that target the 'obesogenic environment', such as community interventions, urban planning, placement of fast food outlets or workplace policies, affect population mean weight and do these effects differ by baseline weight status (underweight, healthy weight, overweight, obesity)? Which of this type of interventions are most effective at reaching low socio-economic groups?
19. Why do some people struggle to maintain motivation during a weight loss attempt?
20. What are the effects of obesity in childhood on outcomes other than physical health e.g. psychological wellbeing, educational achievement, employment, relationship status etc?
21. Does incorporating physical activity into weight loss interventions enhance weight loss? Does this depend upon the frequency and type of physical activity (e.g. high vs low intensity, resistance vs cardiorespiratory activity)?
22. What effect do working hours (e.g. shift work) and/ or a late eating pattern have on body weight and adiposity?
23. Does being physically active mitigate the health consequences of overweight/obesity?

24. Does prolonged psychological stress affect bodyweight? Do interventions that aim to reduce stress increase weight loss in people living with overweight and obesity compared with interventions not addressing stress?
25. Do interventions that address emotional or social overeating lead to weight loss or weight loss maintenance?
26. What is the relationship between emotions and appetite or energy intake?
27. What changes in supermarkets or the wider food industry are effective in promoting healthier diets? Does changing labelling and/ or packaging on foods affect purchasing, consumption and body weight?
28. Do interventions in primary care prevent obesity? What are the effects of offering weight management referrals on the prevalence of obesity?
29. How accurate are existing models of the health consequences of excess weight and the impact of weight loss? Which assumptions are critical in determining the long-term effectiveness and cost-effectiveness of weight loss interventions? What is the impact of weight regain on the incidence of disease and cost-effectiveness of weight loss interventions in these models?
30. How does the brain control food intake and can we use knowledge of these mechanisms to aid weight loss? What are the brain responses to food during weight loss and following weight regain?

Research questions asked by more than 10 people in the first survey – emailed to workshop participants

1. What are the effects of interventions to influence health professionals to support patients with overweight and obesity?
2. Does the gut microbiome have an effect on weight gain or the risk of obesity? Does the gut microbiome influence body composition or fat distribution? Do human genetics influence the gut microbiome? Can a change in gut microbiome aid weight loss and by what mechanisms? Which strains of bacteria are associated with greatest weight loss during a weight loss attempt? What is the effect of prebiotics or probiotics on weight loss/ weight maintenance?
3. Is there a difference in the way that health professionals respond to patients living with obesity compared with people of a healthy weight in terms of investigation, diagnosis and follow up?
4. What are the most effective treatments for obesity in people with severe mental illness? Is integrating weight management into mental healthcare effective in supporting weight loss or weight loss maintenance?
5. Does the mode of delivery e.g. in-person face-to-face, group, digital, telephone influence the weight loss achieved by a weight loss programme?
6. How do the words used to define and describe overweight and obesity affect perceived discrimination as well as emotional and psychological wellbeing in people living with overweight and obesity? Would changing the language reduce the blame culture associated with obesity? What language used in public health messaging about overweight/obesity encourages weight control without increasing stigma towards people living with obesity?
7. What are the advantages and disadvantages of the various methods of quantifying body fat in routine healthcare? What is the association between these measures and risk of obesity-related health outcomes? What are the most appropriate classifications for overweight and obesity in different demographic groups, e.g. ethnicity, age, gender? Can we develop an easy measurement for 'Thin on Outside, Fat on Inside' for use in routine healthcare? What is the association between being 'Thin on Outside, Fat on Inside' and other obesity related outcomes?

8. Is attempting to follow intermittent fasting (including 5:2, every other day diet, etc.) or time restricted eating (e.g. 16:8 or eating between specific hours of the day) effective for weight loss and weight maintenance? Is intermittent fasting or time restricted eating more effective than calorie restriction alone? Are the benefits from intermittent fasting or time restricted eating (e.g. metabolic health, general wellbeing) independent from weight loss? Are there any side effects or health risks from intermittent fasting or time restricted eating?
9. Do people crave or become addicted to certain foods and, if so does this cause obesity? Can treatments reduce pica, food cravings or addiction?
10. Do interventions, including clinical follow-up, after bariatric surgery prevent weight regain? What are the adverse consequences of bariatric surgery and can these be predicted? Why do people living with severe obesity eligible for surgery decide not to proceed with the surgery and do they take up other interventions? What are the psychosocial consequences of bariatric surgery for example, on intimate relationships and eating disorders, and do these vary by social and cultural group?
